# CFM-GP: unified conditional flow matching to learn gene perturbation across cell types

**DOI:** 10.1093/nargab/lqag087

**Published:** 2026-08-10

**Authors:** Abrar Rahman Abir, Sajib Acharjee Dip, Liqing Zhang

**Affiliations:** Bangladesh University of Engineering and Technology, Dhaka 1000, Bangladesh; Department of Computer Science, Virginia Polytechnic Institute and State University, Blacksburg, VA 24061, United States; Department of Computer Science, Virginia Polytechnic Institute and State University, Blacksburg, VA 24061, United States; Department of Health Sciences, Virginia Polytechnic Institute and State University, Blacksburg, VA 24061, United States; Fralin Biomedical Research Institute, Virginia Polytechnic Institute and State University, Blacksburg, VA 24061, United States; FBRI Cancer Research Center, Washington, DC 24016, United States

## Abstract

Understanding how gene perturbations reshape cellular states across diverse contexts is fundamental to functional genomics and therapeutic discovery, yet experimental profiling across all perturbations and cell types remains infeasible. Computational approaches promise scalable inference but often rely on discrete mappings or per–cell-type models that fail to capture continuous and shared biological dynamics. We introduce CFM-GP, a conditional flow-matching framework that learns a continuous vector field transforming control expression profiles into perturbed states, explicitly conditioned on cell type. This unified design models both common regulatory programs and type-specific responses within a single architecture, removing the need to train separate models. Across five single-cell perturbation datasets, CFM-GP consistently outperformed existing methods in predictive accuracy, distributional alignment, and cross-species generalization. The inferred flow trajectories recovered canonical signaling pathways and context-dependent transcriptional cascades, demonstrating mechanistic interpretability. By coupling principled generative dynamics with biological conditioning, CFM-GP offers a scalable foundation for modeling cellular perturbation responses, enabling data-driven exploration of gene function and intervention strategies across heterogeneous cellular systems.

## Introduction

Gene perturbation refers to the deliberate disruption or modification of a gene’s function to observe resulting cellular changes. These interventions provide causal insights into gene regulatory networks by revealing downstream effects on transcription, signaling, and cell fate. Perturbations can be genetic, such as gene knockouts using CRISPR–Cas9 [1, 2], knockdowns via RNA interference (RNAi) [3, 4], or overexpression constructs or chemical, involving small molecules that modulate specific pathways or proteins [5]. The transcriptional response elicited by these perturbations serves as a functional fingerprint, reflecting both direct and indirect regulatory consequences and aiding in the identification of essential genes, pathway architecture, and therapeutic targets [5–8].

The advent of single-cell RNA sequencing (scRNA-seq) has enabled researchers to dissect perturbation responses at unprecedented resolution, capturing heterogeneous and cell-type–specific gene expression changes across complex tissues and conditions [6, 9, 10]. Recent advances in foundation and agentic modeling for single-cell biology further highlight the growing intersection between generative modeling, representation learning, and cellular perturbation analysis [11–13] with recent arXiv works including CellAgent, and scAgent further expanding this direction.

However, realizing the full potential of perturbation-based profiling is hampered by significant experimental bottlenecks. Generating perturbation-response data across a broad range of genes, doses, time points, and especially across diverse cell types is prohibitively expensive and time-intensive [6, 9, 14]. Each additional condition requires laborious experimental design, batch handling, and sequencing, making large-scale perturbation screens infeasible in many biological and clinical settings [15, 16]. As a result, the vast combinatorial space of perturbations and cellular contexts remains sparsely sampled, leaving critical gaps in our understanding of gene function across cell types. This motivates the need for scalable computational models that can generalize perturbation effects across unseen conditions and cell identities [17–19], serving as powerful surrogates for costly experiments and enabling *in silico* hypothesis generation.

Computational models have been developed to predict the transcriptional effects of gene perturbations. Early approaches such as scGen [17], CVAE [20], and trVAE [18] use latent space arithmetic to model expression shifts from control to perturbed states. While effective in some cases, these models assume static perturbation effects and often require retraining for each cell type. scDist [21] offers a statistically robust way to identify affected cell types but cannot generate perturbed profiles or support *in silico* simulations. Methods such as scGen [17] and CVAE [20] struggle to capture complex, nonlinear, and cell-type–specific responses due to their reliance on linear operations and fixed priors. trVAE improves generalization using Maximum Mean Discrepancy (MMD) regularization but lacks explicit cell-type conditioning. scPreGAN [22] enhances distributional realism through adversarial training, yet its dual-GAN setup is hard to train and does not scale well to new or low-data conditions. CoupleVAE [23] improves over scGen and CVAE by learning nonlinear bidirectional mappings between control and perturbed states, offering greater flexibility. However, it relies on separate encoders and decoders, limiting generalization to unseen cell types. More recent approaches such as CPA [24] extend this paradigm to compositional settings, enabling extrapolation across drug dosages and combinations; subsequent multimodal extensions, including MultiCPA (Inecik *et al*., 2022, bioRxiv), further generalize this framework. However, these methods remain constrained by their reliance on discrete latent operations and show limited generalization across diverse biological contexts, especially to unseen cell types. Orthogonal to latent generative approaches, optimal transport (OT)-based models such as CellOT [25] and sc-OTGM (Demir *et al*., 2024, arXiv), have introduced a more principled framework for mapping distributions between control and perturbed populations. These models frame perturbation as a mass-preserving transformation, offering improved distributional fidelity. Yet, most OT-based methods require separate mappings for each cell type and perturbation pair, and often lack an explicit mechanism to model the temporal progression of gene expression dynamics. Recent developments in flow-based generative modeling have shown promise for addressing these gaps. Flow matching techniques learn time-dependent vector fields that transform source to target distributions in a continuous, simulation-free manner. While models such as CellFlow [26] have applied flow matching to simulate cell phenotypes under perturbation, they do not explicitly incorporate the cell type as a conditioning factor and are not optimized for perturbation prediction across diverse biological settings.

To overcome these limitations, we introduce CFM-GP, a unified, conditional flow-matching framework for predicting gene perturbation effects across cell types. CFM-GP models the transformation from unperturbed to perturbed gene expression profiles as a continuous trajectory in the expression space, governed by a neural vector field conditioned on the cell type [27, 28]. This enables the model to capture cell type–specific dynamics within a single shared architecture, eliminating the need for retraining on individual cell types. The novel vector field learns time-dependent perturbation trajectories between paired control and perturbed single-cell profiles, conditioned on both cell identity and time step. By conditioning on cell type embeddings, CFM-GP generalizes across cellular identities, supporting broad application without the need for cell-type–specific models. We benchmark CFM-GP on five biologically diverse datasets including SARS-CoV-2 infection [29], IFN-$\beta$ stimulation, glioblastoma drug treatment, systemic lupus erythematosus, and cytokine-driven progenitor fate transitions from the Statefate collection. Our method consistently outperforms baseline models across key evaluation metrics, including $R^2$ [30], MMD [31], and Spearman correlation [32], capturing both global distributional structure and fine-grained gene expression dynamics. Through gene set enrichment analysis (GSEA) [33], we demonstrate that CFM-GP recovers biologically meaningful pathway activity, validating its predictions at a systems level. We also show that CFM-GP successfully transfers learned perturbation dynamics across species boundaries highlighting its utility in translational and comparative genomics.

## Materials and methods

### Problem formulation

**Figure 1.**
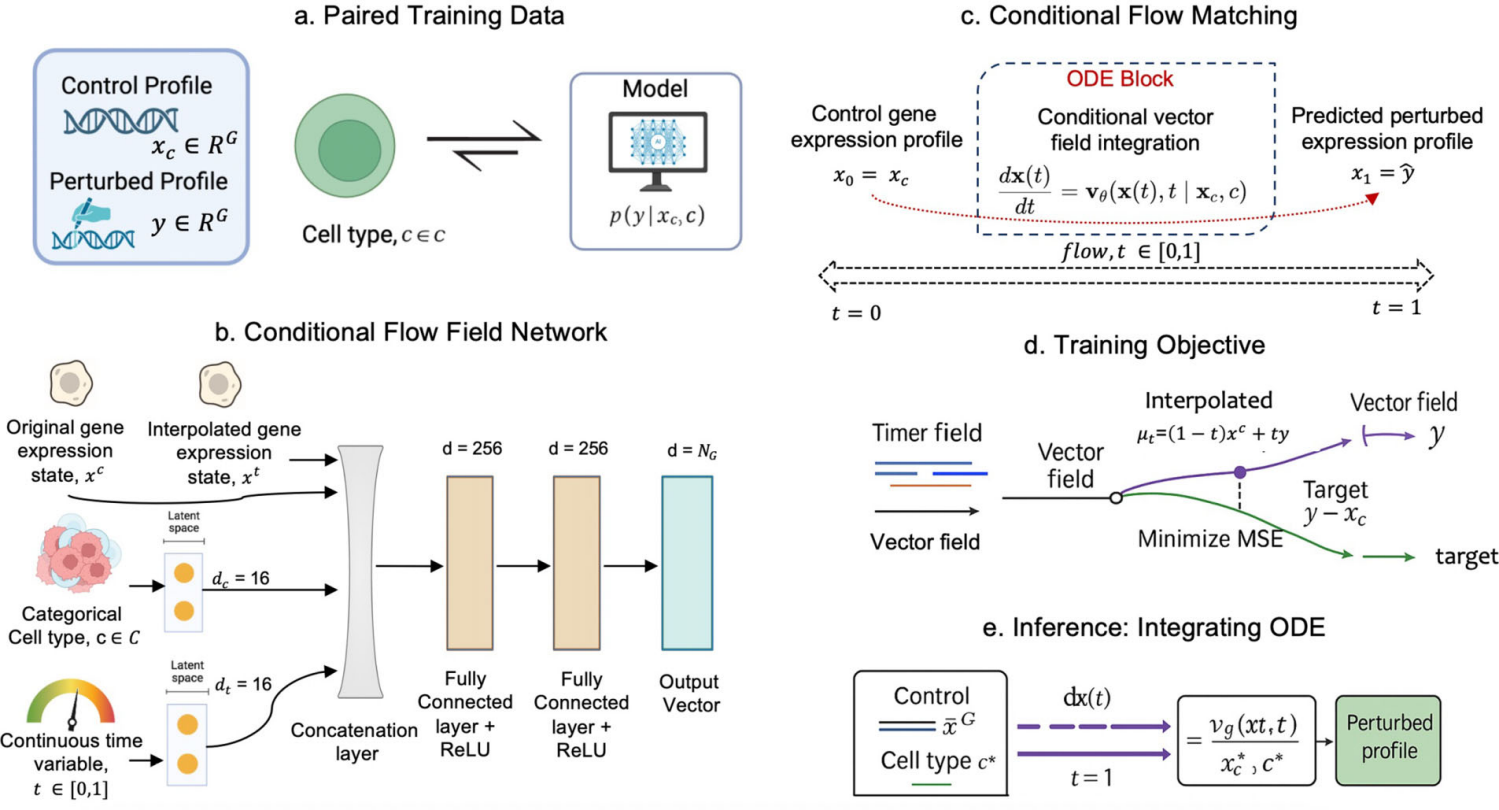
Overview of the CFM-GP modeling framework. (**A**) During training, the model receives paired gene expression profiles of individual cells in control and perturbed conditions, along with their associated cell type labels. (**B**) A conditional vector field network $ \mathbf {v}_\theta (\mathbf {x}(t), t \mid \mathbf {x}_c, c)$ is parameterized by a neural network, where each time step receives the interpolated state $ \mathbf {x}(t)$, original control state $ \mathbf {x}_c$, cell type embedding $ c$, and time embedding $ t$. (**C**) CFM drives the learning of continuous trajectories between the control and perturbed states by matching the learned vector field to the ground-truth direction. (**D**) The model is trained to minimize the mean squared error between the predicted and true velocity vectors across random interpolations between control and perturbed states. (**E**) At inference, the trained vector field is integrated as an ODE from $ t = 0$ to $ t = 1$, starting from a new control profile $ \mathbf {x}_c$ and cell type $ c$, to produce a predicted perturbed profile $ \hat{\mathbf {y}}$.

Modeling the effects of gene perturbation at single-cell resolution is a central challenge in systems biology. Given the control (unperturbed) gene expression profile $\mathbf {x}_c \in \mathbb {R}^G$ of an individual cell and its corresponding cell type $c \in \mathcal {C}$, our objective is to predict the perturbed gene expression profile $\mathbf {y} \in \mathbb {R}^G$ following a perturbation shown in Fig. 1. Here, $G$ denotes the number of genes, and $\mathcal {C}$ is the set of cell types under study. Formally, we aim to learn the conditional distribution: $p(\mathbf {y} \mid \mathbf {x}_c, c),$

where $p$ models the distribution of perturbed gene expression conditioned on both the control expression and cell type. Traditional models for gene perturbation typically learn the distribution $p(\mathbf {y} \mid \mathbf {x}_c)$ separately for each cell type. As a result, they require training and maintaining a distinct model for every cell type $c \in \mathcal {C}$, which greatly limits scalability and cross-cell-type generalization. In contrast, our approach seeks to directly model $p(\mathbf {y} \mid \mathbf {x}_c, c)$ with a single unified model, thereby enabling joint learning across all cell types and more efficient utilization of data.

### Conditional flow matching

We propose to model the conditional transformation from control to perturbed gene expression via conditional flow matching (CFM), which learns a continuous-time vector field parameterized by a neural network (Lipman *et al*., 2022, arXiv). This vector field describes the transformation of $\mathbf {x}_c$ to $\mathbf {y}$, explicitly conditioned on the cell type $c$.

Let $v_\theta (\mathbf {x}, t \mid \mathbf {x}_c, c)$ denote a neural vector field parameterized by $\theta$, which governs the flow from control to perturbed expression over a continuous time variable $t \in [0, 1]$. The transformation is defined as an ordinary differential equation (ODE):


(1) \begin{eqnarray*}
\frac{d\mathbf {x}(t)}{dt} = v_\theta (\mathbf {x}(t), t \mid \mathbf {x}_c, c), \qquad \mathbf {x}(0) = \mathbf {x}_c.
\end{eqnarray*}

The goal is to find $v_\theta$ such that integrating the flow from $t=0$ to $t=1$ transports the control expression $\mathbf {x}_c$ to a sample drawn from the perturbed distribution $p(\mathbf {y} \mid \mathbf {x}_c, c)$.

#### Training objective

During training, we assume access to paired observations $\lbrace (\mathbf {x}_c^{(i)}, \mathbf {y}^{(i)}, c^{(i)})\rbrace _{i=1}^N$, where $(\mathbf {x}_c^{(i)}, \mathbf {y}^{(i)})$ are control and perturbed gene expression profiles of the same cell type $c^{(i)}$. For each pair, we construct interpolated states:


(2) \begin{eqnarray*}
\boldsymbol{\mu }_t = (1-t)\mathbf {x}_c + t\mathbf {y}, \qquad t \sim \mathcal {U}[0, 1].
\end{eqnarray*}

The target vector field at $\boldsymbol{\mu }_t$ is defined as:


(3) \begin{eqnarray*}
\mathbf {v}_{\mathrm{target}} = \mathbf {y} - \mathbf {x}_c.
\end{eqnarray*}

The model is trained by minimizing the mean squared error between the neural vector field and the ground truth direction, across all interpolated states:


(4) \begin{eqnarray*}
\mathcal {L}(\theta ) = \mathbb {E}_{(\mathbf {x}_c, \mathbf {y}, c), t} \left[ \left\Vert v_\theta (\boldsymbol{\mu }_t, t \mid \mathbf {x}_c, c) - (\mathbf {y} - \mathbf {x}_c) \right\Vert ^2 \right].
\end{eqnarray*}

This objective enables the model to learn a continuous, cell-type-aware transformation from control to perturbed gene expression.

#### Inference

At inference time, given a new control profile $\mathbf {x}_c^{*}$ and cell type $c^{*}$, we simulate the learned vector field to generate a predicted perturbed profile $\hat{\mathbf {y}}$ by integrating the ODE:


(5) \begin{eqnarray*}
\frac{d\mathbf {x}(t)}{dt} = v_\theta (\mathbf {x}(t), t \mid \mathbf {x}_c^{*}, c^{*}), \qquad \mathbf {x}(0) = \mathbf {x}_c^{*}.
\end{eqnarray*}

The predicted perturbed profile is taken as $\hat{\mathbf {y}} = \mathbf {x}(1)$. In practice, the ODE is solved by explicit forward Euler integration: the interval $[0, 1]$ is discretized into uniform steps, and at each step, the state is updated by moving in the direction of the learned vector field. This procedure enables fast, cell-type-specific simulation of gene expression responses to perturbations, using only control-state expression and a single, unified model.

### Data preprocessing, pairing, and split protocol

To ensure fair comparison with prior perturbation prediction methods, we adopted the same preprocessing and train/validation/test splits used in the original benchmark pipelines for each dataset (e.g. COVID-19 and PBMC protocols as used in scGen and CoupleVAE). These protocols define: (i) normalization/log-transformation and gene filtering, (ii) the control and perturbed condition keys, and (iii) a fixed split of cells used by prior work. We follow those splits and report results on the same evaluation partitions.

#### Pair construction

All datasets provide cells in control and perturbed conditions with cell-type annotations. Following prior work, we construct balanced paired sets within each cell type by subsampling to the minimum cell count across conditions (min_n) and forming pseudo-pairs (control, perturbed) used for supervised training of perturbation shift models. This pairing strategy is standard in existing baselines and is used to avoid confounding due to cell-count imbalance.

#### Split integrity and leakage prevention

We use the fixed train/validation/test split defined by the baseline pipelines and keep each constructed (control, perturbed) pseudo-pair within the same split (pairs are never separated across splits). Since the split is defined at the cell level in the benchmark protocol, the task evaluates within-dataset generalization to held-out cells under the same perturbation and cell types, rather than cross-donor or cross-perturbation extrapolation unless explicitly stated (e.g. cross-species experiments).

#### Reproducibility

We have added end-to-end preprocessing scripts to the repository that reconstruct the released tensors from the public raw data. As an example, we include an executable script for the COVID-19 dataset that loads the processed AnnData, constructs balanced pseudo-pairs per cell type, and exports tensors used for training and evaluation. We also provide the exact indices and random seeds used to reproduce the reported splits.

### Sensitivity to euler integration steps

During inference, trajectories are integrated from *t* = 0 to *t* = 1 using explicit Euler integration with 10 uniform steps. The model is trained to predict velocities at randomly sampled intermediate times, resulting in a smooth time-dependent vector field. Empirically, increasing the number of integration steps beyond 10 yields negligible performance improvements across immune cell types (Supplementary Fig. S5). Therefore, we adopt 10-step Euler integration as a computationally efficient and sufficiently accurate inference strategy.

### Implementation details

In our implementation, the conditional vector field $v_\theta$ is parameterized by a fully connected neural network that takes as input the current state, control state, time, and cell type, and outputs a velocity vector in gene expression space. Specifically, the input to the network at each time $t$ consists of: the current interpolated gene expression vector $\mathbf {x}_t \in \mathbb {R}^G$, the original control gene expression vector $\mathbf {x}_c \in \mathbb {R}^G$, an embedding vector for the cell type $c$, and a time embedding for $t$. These vectors are concatenated to form a single input vector. The cell type $c$ is embedded via a learnable embedding layer that maps each categorical cell type index to a $d_c$-dimensional vector (we set $d_c=16$). The continuous time variable $t$ is projected to a $d_t$-dimensional embedding (with $d_t=16$) using a linear layer followed by a ReLU nonlinearity.

The concatenated input $[\mathbf {x}_t, \mathbf {x}_c, \mathrm{Embed}(c), \mathrm{Embed}(t)]$ is passed through a multi-layer perceptron (MLP) comprising three fully connected layers. The first two layers have 256 hidden units each, each followed by a ReLU [34] activation. The final output layer projects the hidden representation back to $\mathbb {R}^G$, matching the dimension of the gene expression space. Formally, the MLP can be described as follows:


(6) \begin{eqnarray*}
h_0 = \mathrm{Concat}(\mathbf {x}_t, \mathbf {x}_c, \mathrm{Embed}(c), \mathrm{Embed}(t))
\end{eqnarray*}

(7) \begin{eqnarray*}
h_1 = \mathrm{ReLU}(W_1 h_0 + b_1)
\end{eqnarray*}

(8) \begin{eqnarray*}
h_2 = \mathrm{ReLU}(W_2 h_1 + b_2)
\end{eqnarray*}

(9) \begin{eqnarray*}
v_\theta (\mathbf {x}_t, t \mid \mathbf {x}_c, c) = W_3 h_2 + b_3,
\end{eqnarray*}

where $W_i$ and $b_i$ denote the weights and biases of each layer. Finally, the model was trained for 50 epochs using the Adam [35] optimizer with a learning rate of $1 \times 10^{-4}$ and mean squared error loss as described previously. Mini-batch stochastic gradient descent was employed with a batch size of 32 samples. All experiments were conducted five times and on a cluster equipped with four NVIDIA RTX A4500 GPUs. Training time comparisons across methods are provided in Supplementary Table S11, highlighting the efficiency of CFM-GP.

Statistical significance of performance differences between CFM-GP and baselines was assessed using paired two-sided tests across species transfers (and across cell types within transfers when applicable). Reported *P*-values are included in the revised Results and figure captions. Statistical significance was defined as *P*$< $ 0.05. Phylogenetic divergence times between species were obtained from the TimeTree database [36].

### Evaluation metrics

We evaluate predictive performance using complementary metrics that assess accuracy, distributional alignment, and robustness. First, we report the coefficient of determination (R$^2$) to measure the proportion of variance in gene expression explained by the model. While R$^2$ captures global variance alignment, it may be influenced by overall expression variability. To directly quantify absolute prediction error, we additionally compute mean absolute error (MAE) across genes. MAE provides a scale-sensitive measure of deviation between predicted and observed expression levels and mitigates potential variance-driven inflation effects in R$^2$. To evaluate distributional consistency between predicted and true expression profiles, we compute MMD. MMD assesses whether the predicted and ground-truth distributions match in feature space, capturing higher-order structural differences beyond pointwise error. We also report Spearman correlation to assess rank-level agreement across genes. Together, these metrics provide a comprehensive assessment of variance explained, absolute error, and distributional alignment.

### Hyperparameters settings

For transparency and reproducibility, we provide a complete list of the key hyperparameters used in training and evaluation in Supplementary Table S13. These include settings for the data loaders, model architecture, optimization, and evaluation pipeline. Most parameters follow standard defaults (e.g. Adam optimizer with learning rate $1 \times 10^{-4}$, batch size of 32, and mean squared error loss), while architecture-specific dimensions (e.g. 16-dimensional embeddings for cell type and time, 256 hidden units per layer) were chosen based on preliminary validation performance. Input dimensionality and the number of cell types are automatically inferred from each dataset. The evaluation protocol is likewise standardized, employing ten Euler steps for trajectory integration, RBF kernels for MMD computation, and the top 100 differentially expressed genes for cell-type–specific $R^2$ evaluation. All these hyperparameters were kept consistent across all experiments unless explicitly stated otherwise, ensuring comparability across datasets and models. Hardware details, training epochs, and checkpointing procedures are described in the Materials and methods section, while the full parameter values can be found in Supplementary Table S13.

## Results

### Unified modeling across cell types by conditioning gene perturbation prediction on cell types

Predicting gene perturbation responses across diverse cell types is challenging due to the highly heterogeneous and context-specific nature of transcriptional dynamics. Existing approaches often require separate models for each cell type or fail to explicitly incorporate cell identity, limiting their ability to generalize to unseen cellular contexts. CFM-GP addresses this gap by treating cell type as an explicit conditioning variable within a unified flow-matching framework, enabling a single model to learn shared perturbation principles while preserving cell-type–specific nuances.

In CFM-GP, the transformation from control to perturbed gene expression is formulated as a continuous trajectory in expression space, parameterized by a conditional neural vector field (Fig. 1). At each integration step, the vector field receives four inputs: the current interpolated expression state, the original control state, a learnable cell type embedding, and a continuous time embedding. This conditioning scheme allows the model to capture both the global shared components of perturbation dynamics and the finer cell-type–specific deviations that distinguish one population’s response from another. By integrating the learned vector field as an ODE from $t = 0$ (control) to $t = 1$ (perturbed), CFM-GP produces predictions that remain coherent across diverse cell types while accurately reflecting the unique transcriptional shifts associated with each type. This unified cell-type–aware formulation eliminates the need to train separate models per cell type, improves the utility of data by leveraging the shared structure among populations, and facilitates generalization to contexts not seen during training.

**Figure 2.**
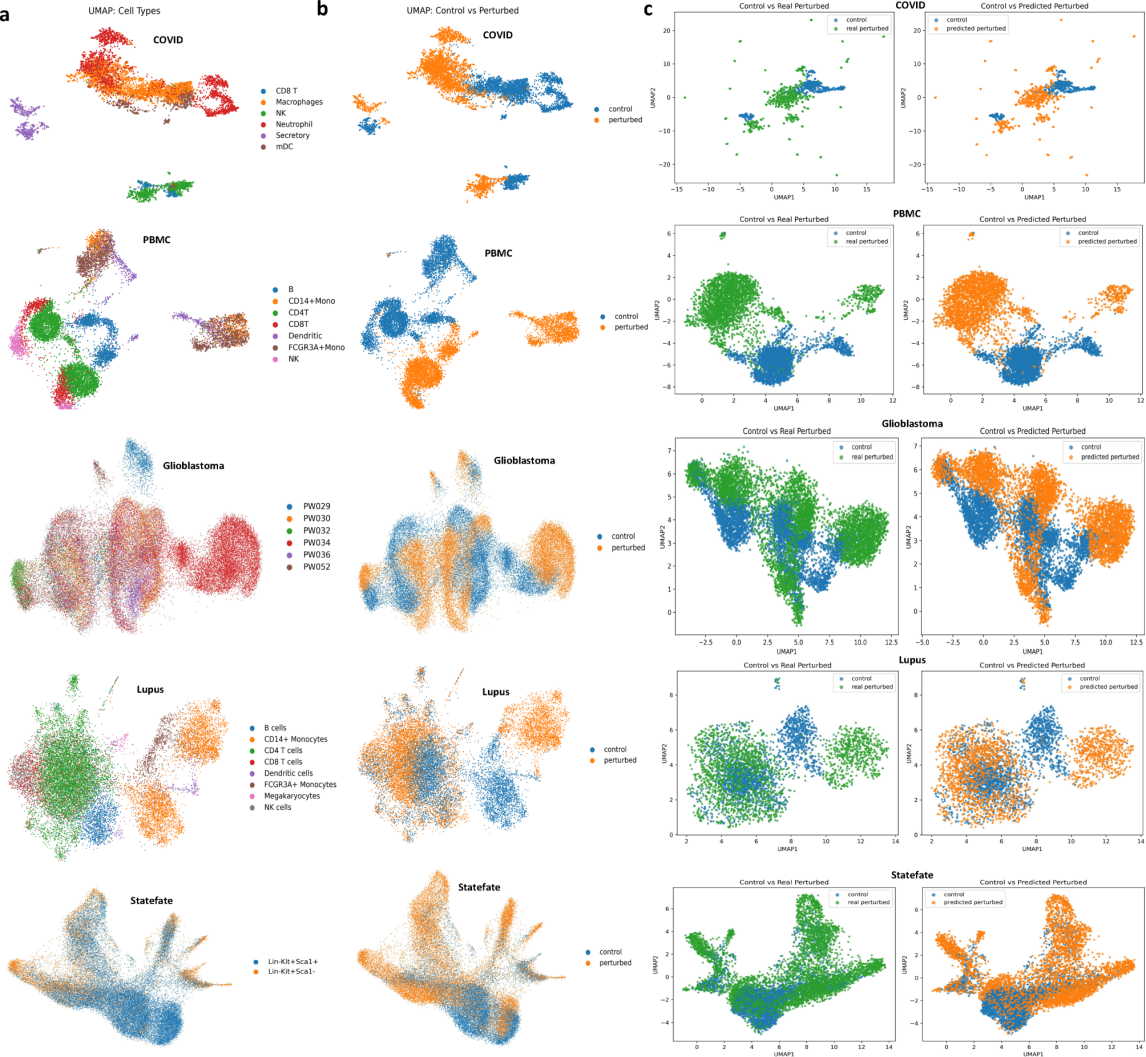
UMAP visualizations illustrating CFM-GP’s ability to preserve cell population structure and accurately reproduce perturbation-induced distribution shifts across multiple datasets. (**A**) UMAP visualization of ground-truth gene expression profiles across five datasets (COVID, PBMC, Glioblastoma, Lupus, Statefate), showing distinct cell population structures. (**B**) UMAP visualization of CFM-GP–predicted gene expression profiles for the same datasets, preserving the clustering patterns observed in the ground truth. (**C**) UMAP comparison of control versus real perturbed cells (left) and control versus predicted perturbed cells (right), showing that CFM-GP predictions closely match the ground-truth perturbation distribution.

The benefits of this design are evident in the UMAP visualizations (Fig. 2) [37, 38]. Ground-truth profiles (Fig. 2A) display well-separated cell-type clusters across diverse datasets, while CFM-GP predictions (Fig. 2B) faithfully preserve these boundaries, capturing both the intra-cell-type structure and inter-cell-type relationships. Furthermore, direct comparisons of control versus real perturbed profiles and control versus predicted perturbed profiles (Fig. 2C) show that the predicted perturbation distributions closely match the true ones, demonstrating that CFM-GP not only maintains overall population structure but also accurately reproduces biologically meaningful perturbation-induced shifts. These qualitative results highlight the model’s ability to unify learning across heterogeneous cell types without sacrificing the specificity of perturbation effects. All datasets consist of strictly paired control and perturbed single-cell profiles (50% each; see Supplementary Note S1. Datasets and Preprocessing and Supplementary Tables S1–S10). Thus, the stronger separation observed in COVID and PBMC reflects larger perturbation-induced transcriptional shifts rather than differences in perturbation proportion. The UMAP visualizations use the same benchmark preprocessing and fixed splits adopted from prior perturbation prediction baselines to ensure comparable evaluation across methods.

### CFM-GP demonstrates strong predictive accuracy across five datasets as measured by *R*^2^ and mean absolute error

We evaluated the predictive accuracy of gene perturbation responses using *R*² values [30] across multiple datasets, comparing our proposed method, CFM-GP, against the baseline method CoupleVAE, as well as benchmark models including scDist, scPreGAN, trVAE, scGen, and CVAE. *R*² scores, quantifying the proportion of variance in gene expression explained by the predictions, were calculated for both the entire gene set (“All Genes”) and the top 100 differentially expressed genes (“Top 100 DEGs”).

**Figure 3.**
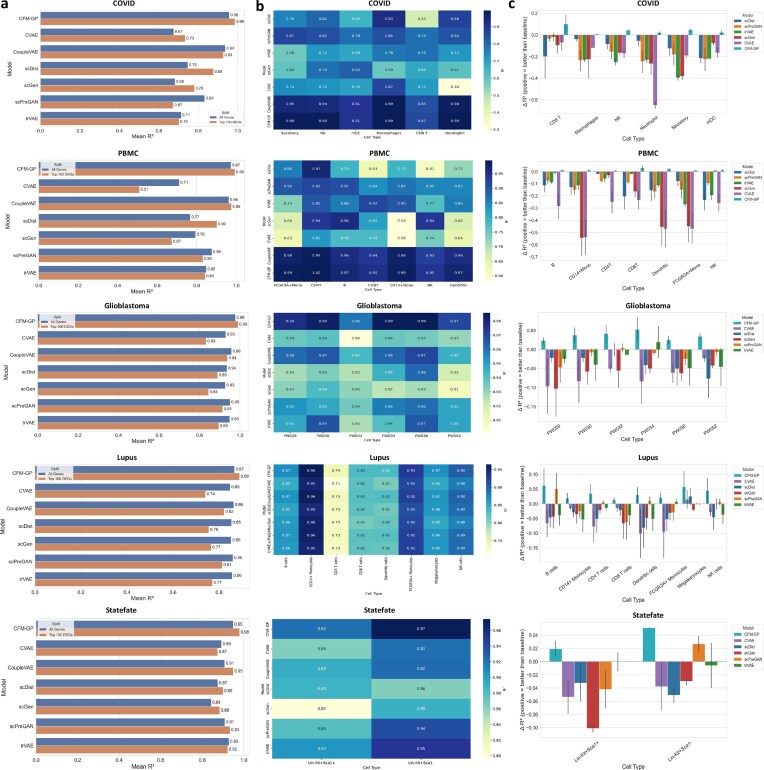
Comparison of predictive performance (*R*^2^) across five datasets COVID-19, PBMC, Glioblastoma, Lupus, and Statefate between CFM-GP and existing baseline models. Panel (**A**) shows absolute *R*^2^ values per model and cell type. Panel (**B**) presents a heatmap of *R*^2^ values across all datasets and cell types. Panel (**C**) illustrates the performance improvement of CFM-GP relative to CoupleVAE ($\Delta R^2$).

The COVID dataset encompasses six distinct cell types: Secretory, NK, mDC, Macrophages, CD8 T, and Neutrophil. Across all these cell types, CFM-GP consistently demonstrated superior predictive accuracy compared to all benchmarking methods. For predictions involving all genes, CFM-GP achieved notably high *R*² scores, ranging from 0.865 in CD8 T cells to 0.995 in Macrophages (Fig. 3A and B). Relative to the baseline method CoupleVAE, CFM-GP showed consistent improvements across all cell types (Fig. 3C). A summary of these results is presented in Table 1, with complete per-cell type results for additional datasets provided in Supplementary Tables S16–S19. The most substantial improvement was observed in NK cells, where CFM-GP achieved an *R*² of 0.987 compared to CoupleVAE’s 0.94, reflecting a 5% increase in predictive accuracy. The smallest observed improvement occurred in mDC cells, with a modest increase from 0.91 to 0.915 (+0.5%). When analyzing only the top 100 DEGs, the performance gap widened considerably in favor of CFM-GP. *R*² scores surpassed 0.994 across all cell types, reflecting particularly pronounced predictive enhancements compared to CoupleVAE. For example, CD8 T cells exhibited the most significant improvement, where CFM-GP achieved an *R*² of 0.955, marking a 24% increase over CoupleVAE’s 0.77. Similarly, substantial gains were observed in Secretory cells (from 0.94 to 0.995, +5.8%) and NK cells (from 0.96 to 0.997, +3.8%). The smallest improvement in this subset was observed in Macrophages, where CoupleVAE’s already high baseline of 0.99 was marginally improved to 0.999 (+0.9%). Comparable trends were observed across PBMC, Glioblastoma, Lupus, and Statefate datasets (see Supplementary Tables S16–S19).

**Table 1.** *R* ^2^ predictive accuracy across COVID-19 cell types for All Genes and Top 100 DEGs. Rows represent models; columns indicate cell types. Percentage improvements for CFM-GP over CoupleVAE are shown in the final rows

Model (Split)	Secretory	NK	mDC	Macrophages	CD8 T	Neutrophil
scDist (All Genes)	0.76	0.81	0.65	0.93	0.45	0.88
scPreGAN (All Genes)	0.87	0.85	0.79	0.85	0.79	0.84
trVAE (All Genes)	0.58	0.72	0.69	0.78	0.79	0.72
scGen (All Genes)	0.60	0.79	0.82	0.59	0.68	0.61
CVAE (All Genes)	0.74	0.73	0.70	0.87	0.71	0.30
CoupleVAE (All Genes)	0.95	0.94	0.91	0.99	0.85	0.98
**CFM-GP (All Genes)**	**0.9861**	**0.9875**	**0.9147**	**0.9946**	**0.8651**	**0.9928**
scDist (Top 100 DEGs)	0.88	0.92	0.76	0.97	0.77	0.96
scPreGAN (Top 100 DEGs)	0.66	0.74	0.60	0.66	0.76	0.62
trVAE (Top 100 DEGs)	0.52	0.68	0.71	0.74	0.79	0.77
scGen (Top 100 DEGs)	0.53	0.78	0.87	0.94	0.75	0.81
CVAE (Top 100 DEGs)	0.77	0.83	0.81	0.87	0.77	0.35
CoupleVAE (Top 100 DEGs)	0.94	0.96	0.93	0.99	0.77	0.97
**CFM-GP (Top 100 DEGs)**	**0.9949**	**0.9967**	**0.9697**	**0.9991**	**0.9552**	**0.9992**
*% Imprv (All Genes)*	+3.81%	+5.05%	+0.81%	+0.46%	+1.77%	+1.30%
*% Imprv (Top 100 DEGs)*	+5.88%	+3.65%	+4.27%	+0.91%	+24.06%	+2.90%

Comparing CFM-GP with other benchmark methods (scDist, scPreGAN, trVAE, scGen, and CVAE) further underscores its robust performance. For example, in CD8 T cells, CFM-GP significantly outperformed CVAE and scDist by 18.5%, improving from approximately 0.77 to 0.955. In Macrophages, where all methods generally performed well, CFM-GP still notably enhanced predictive accuracy, raising the benchmark from approximately 0.97 achieved by scDist to a near-perfect *R*² score of 0.999. The PBMC dataset includes seven immune cell types: FCGR3A+ Monocytes, CD4 T cells, B cells, CD8 T cells, CD14+ Monocytes, NK cells, and dendritic cells. CFM-GP again outperformed all benchmarking methods across every cell type. When using all genes, CFM-GP achieved *R*² scores ranging from 0.946 (NK) to 0.995 (CD4 T), surpassing CoupleVAE in all cases. The relative improvements in *R*² over CoupleVAE were modest but consistent, with the largest improvement observed in NK cells (+1.69%) and the smallest in CD4 T cells (+0.55%). The predictive gains became more substantial when focusing on the top 100 DEGs. CFM-GP reached near-perfect *R*² values across all cell types, including 0.999 for CD4 T and 0.998 for FCGR3A+ Monocytes. CD8 T cells saw the most pronounced improvement, increasing from 0.95 to 0.995 (+4.71%). B cells and dendritic cells also showed marked increases (+1.43% and +2.74%, respectively). Even in cell types where CoupleVAE performed well, such as FCGR3A+ Mono and CD14+ Mono, CFM-GP provided additional improvements. In contrast, models like CVAE and scGen frequently underperformed, particularly in monocyte populations, where *R*² values for top DEGs were as low as 0.30–0.40.

In the Glioblastoma dataset, CFM-GP demonstrated improvements over CoupleVAE in every sample, with gains ranging from +1.59% (PW036) to +2.66% (PW052). The performance gap widened when evaluating the top 100 DEGs, where CFM-GP outperformed CoupleVAE by as much as +9.36% in PW034. Notably, CFM-GP maintained an *R*² above 0.985 in all samples for the All Genes setting, while other methods including trVAE and CVAE showed variable performance, particularly in more heterogeneous samples such as PW032. In the lupus dataset, which comprises immune cell profiles from lupus patients under Interferon beta, IFN-$\beta$ stimulation, CFM-GP consistently outperformed all baseline methods across eight evaluated cell types. In the all-genes setting, the improvements over CoupleVAE were modest but uniform, ranging from +0.40% in NK cells to +0.74% in CD8 T cells, indicating consistent generalization across inflammatory immune states. More pronounced gains were observed in the top 100 DEGs condition, where CFM-GP achieved the highest *R*² scores in all cell types. Notably, it delivered a +22.30% improvement in Megakaryocytes, a population where most competing models, including CoupleVAE and CVAE, struggled to exceed *R*² scores of 0.55. CFM-GP also achieved near-perfect predictive accuracy in CD14+ Monocytes (*R*² = 0.994) and FCGR3A+ Monocytes (*R*² = 0.984), outperforming both deep generative and deterministic baselines. Despite involving only two progenitor subpopulations, the Statefate data revealed nuanced differences in how models captured cytokine-induced transcriptional shifts. CFM-GP stood out for its ability to generalize across both early stem-like (*Lin-Kit+Sca1+*) and more committed (*Lin-Kit+Sca1-*) cells. In the all-genes setting, it delivered improvements of +3.5% and +5.5% *R*^2^ over CoupleVAE, with further refinement observed in the top 100 DEGs condition. Notably, the Sca1- population often more transcriptionally variable saw the greatest benefit, with CFM-GP achieving near-perfect *R*^2^ (0.996), outperforming all baselines. To complement $R^2$, we evaluated MAE across all datasets and cell types to directly quantify absolute prediction error (Supplementary Fig. S6 and Supplementary Table S30). Across COVID-19, PBMC IFN-$\beta$, lupus, glioblastoma, and Statefate datasets, MAE values remain consistently low and follow trends aligned with improvements observed in $R^2$. This confirms that the performance gains are not driven by variance inflation but reflect genuine reductions in gene-level prediction error.

We further assessed numerical stability by varying the number of Euler integration steps during inference (10–100 steps; Supplementary Fig. S5). MAE remains stable across integration depths, with only negligible changes observed at higher step counts (typically limited to the fourth decimal place). These results demonstrate that CFM-GP achieves both low absolute error and numerical robustness, and that 10-step Euler integration provides a sufficient and computationally efficient approximation of the learned trajectory.

### CFM-GP accurately captures distributional similarity with low MMD across diverse datasets

In addition to gene-level accuracy metrics, we evaluated how closely the predicted gene expression distributions matched the real perturbed distributions using MMD. As a kernel-based statistical measure of distributional distance, MMD provides a complementary assessment to pointwise metrics such as *R*^2^. Lower MMD values indicate better alignment between the predicted and observed perturbation distributions, reflecting the biological plausibility of a model’s generative capacity.

**Figure 4.**
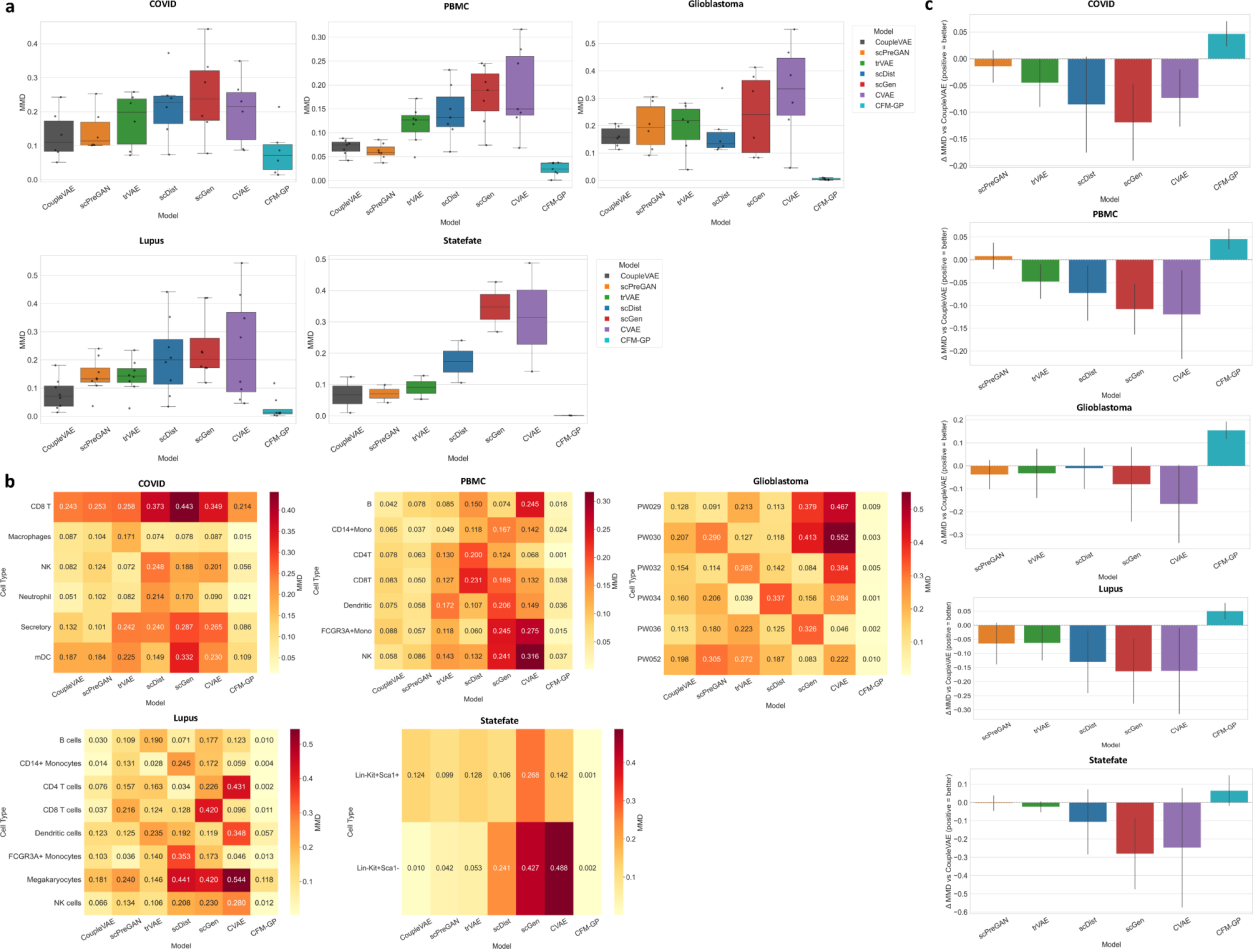
Comparison of distributional similarity between predicted and real gene expression across models using MMD. (**A**) Boxplots showing MMD values for each model across five datasets (COVID, PBMC, Glioblastoma, Lupus, Statefate); lower values indicate better alignment. (**B**) Heatmaps of MMD scores per model and cell type, highlighting CFM-GP’s consistent performance across diverse biological settings. (**C**) Bar plots showing average MMD improvement ($\Delta$MMD) of each model relative to CoupleVAE, with CFM-GP consistently achieving the largest reductions in distributional divergence.

Across all six COVID-19 cell types, CFM-GP achieved the lowest MMD scores compared to all baseline methods (Fig. 4A and Supplementary Table S20). For example, CFM-GP reduced MMD by over 83% relative to CoupleVAE in macrophages and by 58% in neutrophils. Improvements were also notable in NK cells (31%) and secretory cells (35%), where alternative models such as CVAE and scGen showed substantially higher divergence. Although trVAE slightly outperformed CoupleVAE in NK cells, CFM-GP remained the most consistent performer across all subsets.

The PBMC dataset further demonstrates CFM-GP’s strength in capturing accurate perturbation distributions across immune subtypes. As shown in Fig. 4A and Supplementary Table S21, CFM-GP achieved the lowest MMD values in all seven evaluated cell types. In B cells and CD8 T cells, MMD was reduced by over 50% relative to CoupleVAE. The largest reduction was observed in CD4 T cells, where CFM-GP nearly eliminated distributional divergence (MMD = 0.0008, +98.9% improvement). Figure 4B highlights broad divergence in baseline methods, particularly CVAE and scGen, whereas CFM-GP maintained a narrow and consistent distribution across all subsets. Additionally, the delta-MMD plot (Fig. 4C) shows that CFM-GP consistently yields lower MMD than CoupleVAE, unlike several other models that show cell-type–dependent variation or even worse performance.

Distributional evaluation on glioblastoma patient samples confirmed the robustness of CFM-GP across individual-level heterogeneity. As detailed in Supplementary Table S22, CFM-GP achieved the lowest MMD scores across all six samples, with reductions ranging from +93% to +99% over CoupleVAE. In PW034, the model reduced the MMD from 0.160 to 0.0009, a 99.5% improvement, while even more modest samples like PW029 showed over 93% reduction. Compared to competing methods, including trVAE, CVAE, and scGen, which exhibited substantial variance and outlier behavior (Fig. 4A), CFM-GP maintained a tightly clustered low-discrepancy range. Heatmaps (Fig. 4B) further illustrated how CFM-GP uniformly minimized distributional mismatch across all patients, and delta-MMD plots (Fig. 4C) reinforced that these gains were not driven by a single sample but generalized across the cohort.

In the lupus dataset, which captures IFN-$\beta$–stimulated responses in autoimmune patient-derived immune cells, CFM-GP again demonstrated the strongest distributional alignment. According to Supplementary Table S23 and Fig. 4A, CFM-GP consistently achieved the lowest MMD values across all eight cell types. Reductions relative to CoupleVAE were substantial: +97% for CD4 T cells, +87% for FCGR3A+ monocytes, and over +81% for NK cells. Even in more difficult populations such as megakaryocytes, where all models struggled, CFM-GP offered the lowest discrepancy (MMD = 0.1176), outperforming others by a wide margin. The heatmap in Fig. 4B clearly visualizes this trend, with CFM-GP’s column standing out as uniformly light across rows, in contrast to the darker, more variable entries of baseline methods. The delta-MMD summary (Fig. 4C) confirms CFM-GP’s broad and consistent advantage, underscoring its capacity to learn perturbation distributions faithfully even in immunologically dysregulated conditions.

Though composed of only two progenitor compartments, the Statefate dataset revealed sharp contrasts in model fidelity. CFM-GP achieved the lowest MMD in both Lin-Kit+Sca1+ and Lin-Kit+Sca1- populations, with near-complete alignment in the former (MMD = 0.0007, +99.5% over CoupleVAE) (see Supplementary Table S24). Figure 4A highlights this striking contrast: while most models including trVAE and scDist struggled with distributional consistency, CFM-GP’s predictions were tightly concentrated and well-aligned. The corresponding delta-MMD plot (Fig. 4B) shows CFM-GP as the only model yielding consistent positive gains in both subsets. These findings reinforce CFM-GP’s robustness not only in immune perturbation settings but also in developmental transitions involving subtle transcriptional shifts.

### CFM-GP preserves gene expression ordering across multiple datasets

To evaluate whether perturbation models retain the relative structure of gene expression changes, we computed the Spearman rank correlation ($\rho$) between predicted and real gene expression profiles. Unlike *R*^2^, which assesses absolute correspondence, Spearman $\rho$ captures the monotonic relationship between predicted and ground truth gene rankings, an important feature when assessing biological relevance in differential expression and pathway inference tasks.

**Figure 5.**
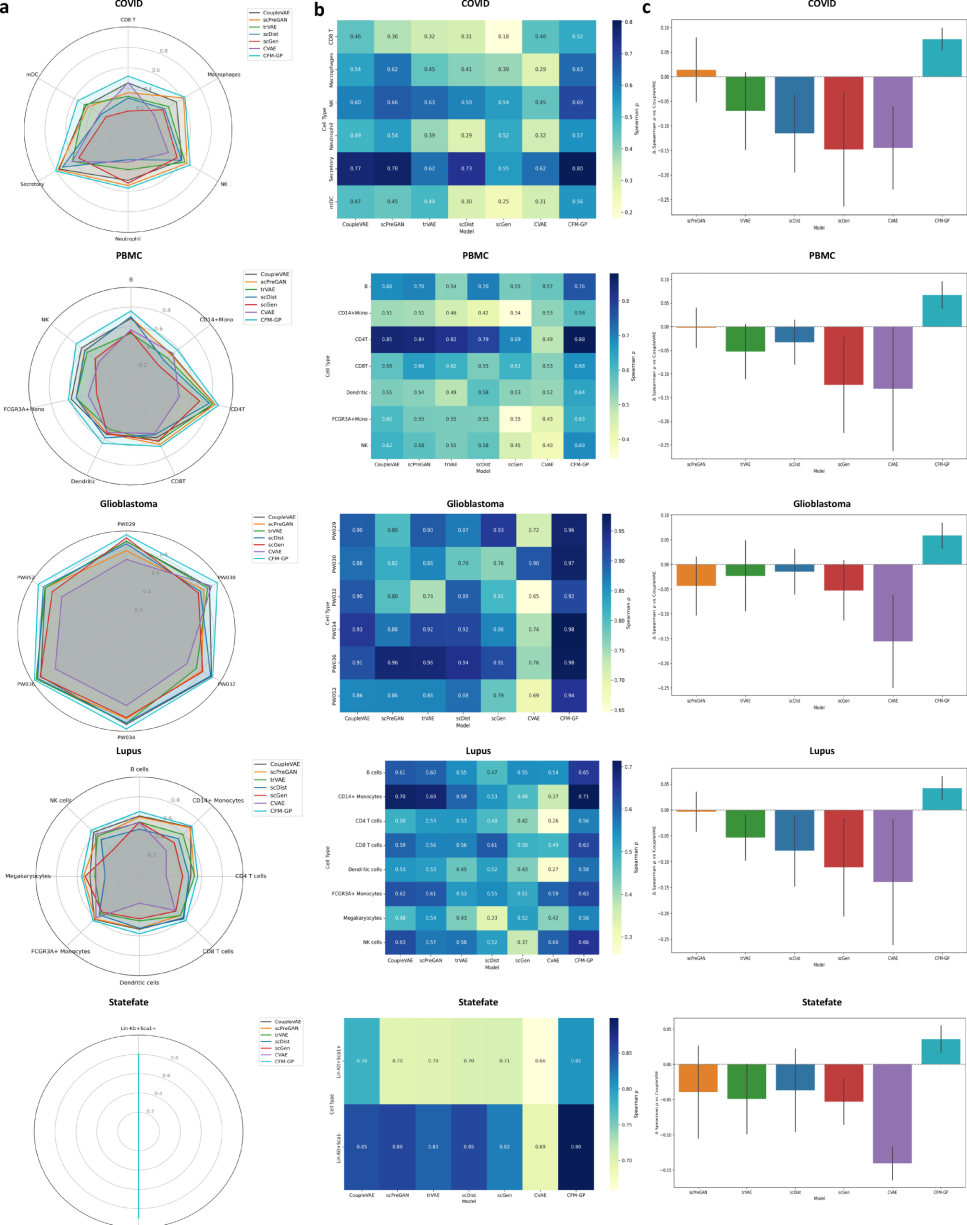
Evaluation of gene ranking preservation using Spearman correlation. (**A**) Radar plots showing average Spearman correlation ($\rho$) across cell types for each model. (**B**) Heatmap of Spearman $\rho$ values across models and cell types, where higher values indicate better consistency in predicted gene expression rankings. (**C**) Mean improvement in Spearman $\rho$ of each model over the baseline (CoupleVAE), evaluated using paired *t*-tests across cell types.

We benchmarked CFM-GP against six competitive models across all five datasets using per-cell-type Spearman correlations [32]. Figure 5 visualizes the performance: radar plots summarize per-model consistency across cell types; heatmaps reveal localized performance variation; and delta plots illustrate each model’s gain over the baseline method (CoupleVAE).

As shown in Supplementary Table S25, CFM-GP consistently achieved the highest Spearman $\rho$ values across all six cell types, indicating that it most accurately preserved the rank ordering of gene expression following perturbation. Compared to the strongest baseline, CoupleVAE, CFM-GP improved Spearman correlation by +14.7% in CD8 T cells, +17.4% in macrophages, and nearly +20% in mDCs. Notably, even in cell types like NK and neutrophils where baselines such as scPreGAN and scGen performed competitively, CFM-GP still delivered the top ranking fidelity. These trends are evident in the radar view (Fig. 5A) and heatmap (Fig. 5B), where CFM-GP shows uniform superiority. The bar chart of delta-Spearman (Fig. 5C) further confirms that CFM-GP provides consistent rank-preserving benefits across cellular contexts.

In the PBMC dataset, CFM-GP outperforms all competing models across all seven cell types, achieving the highest Spearman correlation in each case shown in Supplementary Table S26. While CoupleVAE generally ranked second particularly in CD4T and FCGR3A+ monocytes, its performance lagged further behind in dendritic and CD8T cells, where models like scPreGAN and scDist came closer. The largest relative improvements from CFM-GP were observed in dendritic cells (+18.0%) and CD8T cells (+17.1%), suggesting enhanced sensitivity to subtle ordering shifts in more heterogeneous or transcriptionally variable populations. CD4T cells saw the smallest margin of improvement (+3.9%), possibly due to already high baseline performance from CoupleVAE in this subset. Across models, trVAE and CVAE consistently ranked lowest, often underperforming even scDist, while scPreGAN performed competitively only in B cells.

As shown in Supplementary Table S27, CFM-GP achieved the highest Spearman rank correlation in all six glioblastoma patient samples. The strongest relative gains were observed in PW029 (+7.5%), PW030 (+9.5%), and PW052 (+8.7%), where CFM-GP consistently outperformed both the baseline CoupleVAE and all other methods. Interestingly, while scGen exhibited competitive performance in PW029 (0.933) and trVAE matched or exceeded CoupleVAE in several instances (e.g. PW036), these methods exhibited more variability across patients. For instance, CVAE and scPreGAN had lower and less consistent performance, often trailing by a wide margin. In PW032 and PW034, the improvement of CFM-GP over CoupleVAE was more modest (+1.5% and +5.0%, respectively), likely due to already strong baseline performance.

The lupus dataset, derived from interferon-$\beta$–stimulated patient samples, exhibited broader variability in Spearman correlations across models and cell types. CFM-GP delivered top performance for all eight populations, with the greatest relative gains in megakaryocytes (+16.5%) and CD4 T cells (+13.3%) shown in Supplementary Table S28. These improvements suggest that CFM-GP is particularly robust in transcriptionally noisy or heterogeneous compartments. On the other hand, baseline models like CoupleVAE performed reasonably well in CD14+ monocytes and FCGR3A+ monocytes, narrowing CFM-GP’s margin to under 3%. While trVAE and scPreGAN occasionally came close, such as in B and CD4 T cells, neither consistently ranked second. CVAE and scDist underperformed in nearly all populations.

In the Statefate dataset, which focuses on two hematopoietic progenitor populations, CFM-GP achieved the highest Spearman rank correlation in both Lin-Kit+Sca1+ and Lin-Kit+Sca1- subsets, with modest but consistent gains over the best baselines. While CoupleVAE and scPreGAN delivered relatively strong performance, especially in Lin-Kit+Sca1- CFM-GP still surpassed them, improving correlation by +2.8% and +5.8% respectively (Supplementary Table S29). Interestingly, while differences across models were smaller than in other datasets, CFM-GP’s edge was most apparent in the more transcriptionally complex Lin-Kit+Sca1- population, hinting at its sensitivity to nuanced gene ordering. CVAE trailed significantly in both cell types, and scDist underperformed in Lin-Kit+Sca1+, reaffirming CFM-GP’s ability to preserve rank structure in subtle developmental contexts.

### Pathway analysis reveals strong biological consistency with real data through Normalized Enrichment Scores

Pathway enrichment analysis is commonly used to interpret differential gene expression results in terms of higher-level biological processes. Rather than analyzing individual gene changes, enrichment methods assess whether specific pathways, defined by curated gene sets are statistically overrepresented among the most perturbed genes. This helps link transcriptional variation to known signaling cascades or functional modules. In our study, we applied GSEA using ranked gene lists derived from the predicted and real expression profiles for each cell type. The gene sets were obtained from MSigDB collections, including Hallmark and KEGG pathways. Enrichment was computed using the preranked GSEA method, and Normalized Enrichment Scores (NES) [33] were used to compare predicted versus real pathway activations. This approach enabled a functional-level evaluation of whether CFM-GP accurately recovered the biological programs perturbed in each condition.

To assess whether CFM-GP’s predictions retain functional biological relevance beyond expression-level fidelity, we conducted a pathway enrichment analysis using the top DEGs derived from both real and predicted expression profiles across four datasets (COVID-19, PBMC, Lupus, and Statefate). GSEA was performed independently on each condition using curated hallmark and KEGG pathways, and the resulting NES were used for comparative evaluation.

Supplementary Figures S1– S4 summarizes the NES values for both real and predicted gene expression profiles across cell types and datasets. Visually, CFM-GP demonstrates strong concordance with the NES signatures of the real data, recapitulating key biological signals in both direction and magnitude. In particular, pathways associated with interferon signaling, inflammatory response, and metabolic reprogramming were consistently captured across immune-relevant cell types in COVID-19 and peripheral blood mononuclear cell (PBMC) datasets. Similarly, in the Lupus dataset, IFN-stimulated transcriptional programs were accurately recovered, further supporting the model’s robustness to disease-specific perturbation contexts. For Statefate progenitor compartments, CFM-GP correctly preserved lineage-specific programs linked to stem and progenitor regulation, despite the relatively subtle transcriptional gradients inherent in this developmental dataset.

In the COVID-19 dataset, CFM-GP closely matched real NES profiles in macrophages, neutrophils, and mDCs, accurately recovering pathways such as Toll-like receptor signaling, cytokine interaction, and chemokine signaling with preserved direction and relative magnitude. Minor deviations were observed in predicted NES values for metabolic and apoptotic pathways, though overall trends aligned. In contrast, CD8 T cells showed few enriched pathways and limited recovery, reflecting weak transcriptional signal in this subset (see Supplementary Fig. S1).

In the PBMC dataset, CFM-GP closely tracked real NES scores [33] across major immune subsets, particularly for interferon signaling, cytokine interaction, and antigen presentation in monocytes, dendritic cells, and NK cells. Directionality and magnitude were well preserved, especially in innate compartments. In CD4 and CD8 T cells, pathway concordance was more variable, with some over- or underestimation in metabolic and viral-response pathways, suggesting reduced accuracy in modeling adaptive, cell-specific programs (see Supplementary Fig. S2).

In the Lupus dataset, CFM-GP reliably recapitulated key interferon-driven transcriptional programs across B cells, monocytes, and dendritic subsets hallmarks of systemic autoimmune activation. Predicted NES values closely mirrored real scores for cytokine signaling, antigen presentation, and viral response, with well-matched directionality and only moderate magnitude deviations. Minor discrepancies emerged in more heterogeneous adaptive compartments such as CD4 and CD8 T cells, where pathway-level variation was less consistently captured (Supplementary Fig. S3).

In the Statefate dataset, CFM-GP preserved pathway-level regulation with notable consistency across both $\mathrm{Lin}^-\mathrm{Kit}^+\mathrm{Sca1}^+$ and $\mathrm{Lin}^-\mathrm{Kit}^+\mathrm{Sca1}^-$ compartments, particularly for immune-related and adhesion pathways such as leukocyte migration, phagosome, and CAM signaling. While real NES values showed more polarized activation in certain contexts (e.g. MAPK and NF-$\kappa$B in Sca1$^+$), the predicted scores captured directionality but tended to compress the dynamic range, indicating that the model preserves qualitative trends while smoothing quantitative extremes (Supplementary Fig. S4).

**Figure 6.**
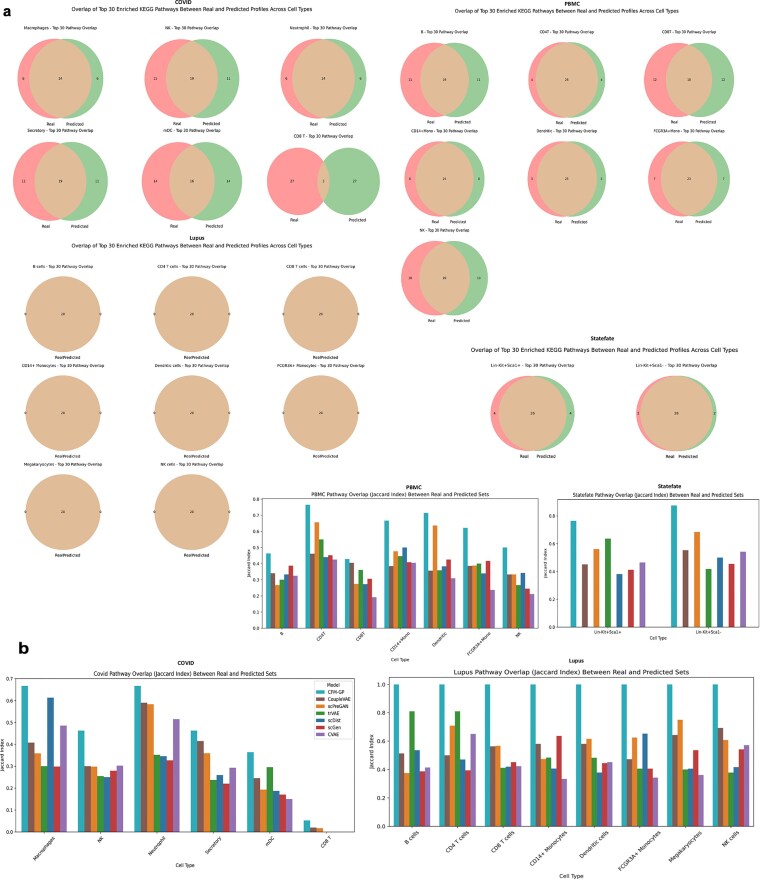
Pathway overlap analysis between real and predicted gene expression responses across four benchmark datasets. (**A**) Venn diagrams showing the overlap between top enriched pathways (based on NES) derived from real and CFM-GP–predicted gene expression profiles. Pathway recovery was complete in lupus, substantial in IFN-$\beta$–stimulated PBMC and COVID-19, and moderate in cytokine-treated progenitors (Statefate), reflecting dataset complexity and developmental variation. (**B**) Bar plots display Jaccard similarity scores across cell types and methods, quantifying how well each model preserved pathway-level responses. CFM-GP consistently shows the highest alignment with ground-truth signatures, particularly in innate immune subsets, indicating improved biological fidelity over competing generative models.

To quantify the overlap between predicted and real pathway hits, we constructed Venn diagrams comparing the top 10 enriched pathways per cell type (Fig. 6A). CFM-GP recovered all biologically enriched pathways in the Lupus dataset, showing perfect overlap with real signals. COVID-19 and PBMC datasets also exhibited substantial intersection, with minor divergence arising from predicted pathways that were plausible but not top-ranked in the real data. In the Statefate dataset, a moderate degree of overlap was observed, likely a reflection of the lower signal-to-noise ratio and fewer pathway-level perturbations typical of progenitor cells.

We used Jaccard similarity to quantify the overlap between predicted and real top-ranked pathways per cell type, computed as the ratio of shared pathways to the union of predicted and ground-truth sets (Fig. 6B). This allowed us to evaluate how well each model preserved biologically meaningful pathway signatures.

In the COVID data set, CFM-GP maintains a strong alignment of the pathway in key innate immune cells such as Macrophages and Neutrophils (Jaccard = 0.67 for both), indicating the robustness to capture infection-induced transcriptional programs. In contrast, all models struggle with adaptive compartments, with CD8 T cells showing near-zero overlap across the board, reflecting limitations in modeling T cell–specific responses under severe perturbation. In the PBMC dataset, CFM-GP consistently outperforms other models across all cell types, achieving the highest Jaccard index in most cases, particularly for CD4T and Dendritic cells. This suggests that its feature-aware design may better capture biological pathway coherence. In contrast, models like CVAE and scGen underperform on immune subsets, indicating limitations in preserving functional gene sets under domain shift. In the Lupus dataset, CFM-GP consistently achieves perfect pathway recovery (Jaccard = 1.0) across all immune cell types, suggesting exceptional robustness in capturing cell-type–specific gene sets under disease perturbation. Meanwhile, other models show variable performance, particularly weaker overlap in monocyte and dendritic subsets, highlighting challenges in modeling pathway integrity for myeloid populations under domain shift. In the Statefate dataset, CFM-GP demonstrates the strongest pathway fidelity, achieving Jaccard scores of 0.76 and 0.88 for Lin$^-$Kit$^+$Sca1$^+$ and Lin$^-$Kit$^+$Sca1$^-$ populations, respectively. Notably, while scPreGAN and trVAE perform moderately well for Lin$^-$Kit$^+$Sca1$^+$ (0.56 and 0.64), their accuracy drops for Lin$^-$Kit$^+$Sca1$^-$, highlighting potential challenges in generalizing across early hematopoietic states.

### CFM-GP exhibits strong out-of-distribution performance on cross-species data

Robust generalization across species is essential for modeling evolutionarily conserved gene perturbation programs and enabling translational inference. To evaluate whether CFM-GP can capture transferable response patterns, we assessed its performance in a cross-species perturbation setting using data from primary mononuclear phagocytes collected across four mammalian species: mouse, pig, rabbit, and rat. All cells were stimulated with lipopolysaccharide (LPS) for 6 h, generating paired gene expression profiles for both control and perturbed states. This setup mirrors biological conditions where different species respond to the same stimulus with both conserved and divergent transcriptional signatures.

We define the cross-species perturbation task as an out-of-distribution (OOD) generalization problem. Specifically, models are trained on all cells from one source species (control + perturbed) and evaluated on held-out target species. This formulation tests whether perturbation dynamics learned in one species can accurately predict gene expression changes in another, given only control data from the target. CFM-GP’s performance was benchmarked against CoupleVAE across all 12 possible source-target combinations. Supplementary Table S31 reports $R^2$ values for both models alongside their relative improvement.

CFM-GP achieved higher predictive accuracy in 9 of the 12 cross-species transfers. The largest gains were observed in transfers from rat$\rightarrow$mouse (+8.1%), rabbit$\rightarrow$mouse (+4.9%), and mouse$\rightarrow$rabbit (+4.5%). These results suggest that CFM-GP captures generalizable perturbation directions that extend across species boundaries. However, in three settings, most notably pig$\rightarrow$rabbit (–6.4%) CoupleVAE marginally outperformed CFM-GP, likely due to subtle alignment mismatches or limited divergence in perturbation signatures where latent alignment suffices.

**Figure 7.**
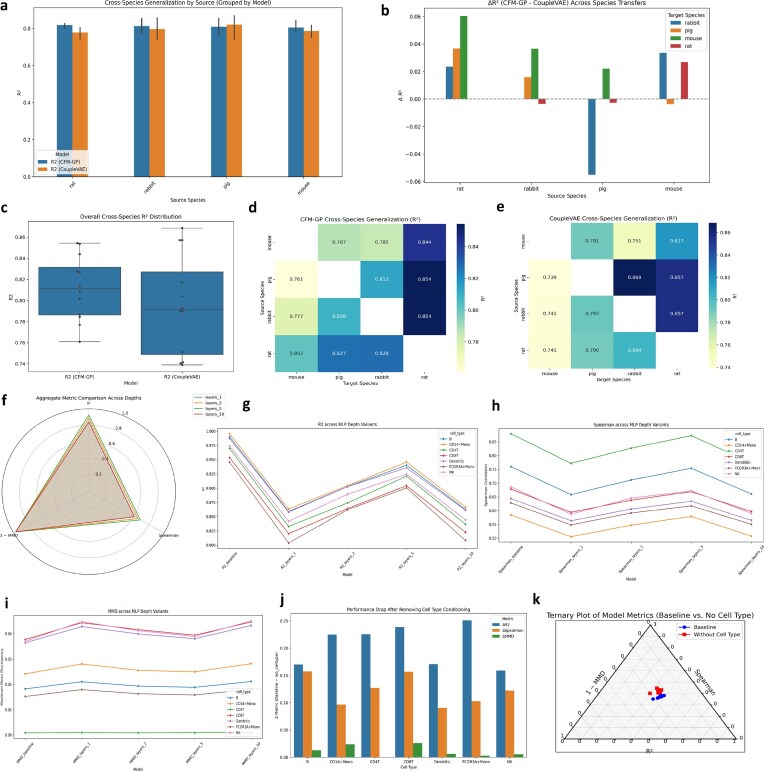
Cross-species generalization performance comparison between CFM-GP and CoupleVAE. (**A**) Grouped barplot showing mean $R^2$ per source species across all targets. (**B**) $\Delta R^2$ barplot comparing CFM-GP and CoupleVAE across individual source-target species pairs. (**C**) Boxplot showing the distribution of $R^2$ values aggregated over all transfers. (**D** and **E**) Heatmaps showing per-pair $R^2$ values for CFM-GP and CoupleVAE respectively, where rows represent source species and columns represent target species. (**F**) Aggregate performance of CFM-GP across MLP depths (baseline excluded), with the 5-layer model best among ablated versions, highlighting medium-depth architectures (3–5 layers) for balanced accuracy and generalization. (**G**) R$^2$ scores per cell type across MLP depths, with baseline model retaining the highest predictive accuracy. (**H**) Spearman correlation per cell type across depths, highlighting superior rank preservation by medium-depth architectures. (**I**) Line plot to show MMD across MLP depths. (**J**) Performance drop across cell types without cell type conditioning, with largest declines in R$^2$ and Spearman correlation. (**K**) Ternary plot showing metric trade-offs with and without cell type conditioning, revealing systematic shifts toward $1{-}\mathrm{MMD}$ and away from *R*$^2$ and Spearman, consistent with panel (J).

Figure 7A shows model-averaged $R^2$ scores grouped by source species. Across cross-species transfers, CFM-GP showed consistent improvements over baseline methods; paired testing confirmed statistically significant improvements in selected comparisons (*P*$< $ 0.05) for each species, indicating robustness to domain shift regardless of origin. CFM-GP consistently maintains higher or comparable means for each species, indicating robustness to domain shift regardless of origin. Interestingly, while CoupleVAE trails by a few percentage points in most settings, the margin is larger when the source is rat or mouse species with more consistent perturbation signals, possibly due to CFM-GP’s ability to model continuous transformation flows rather than rely on latent sampling alone. Figure 7B presents a detailed $\Delta R^2$ comparison between the two models. Each bar shows the relative improvement (or decline) of CFM-GP over CoupleVAE for a specific source-target pair. Most bars lie above zero, reflecting CFM-GP’s stronger transfer performance. Notably, the largest improvements occur when the source species is phylogenetically more distant from the target (e.g. rat$\rightarrow$mouse), where modeling continuous conditional dynamics may provide an advantage over static latent interpolation. Figure 7C summarizes the overall $R^2$ distribution across all 12 transfers. The median for CFM-GP is visibly higher than CoupleVAE, and the spread is tighter. This suggests that not only does CFM-GP achieve stronger mean performance, but it is also more consistent across diverse conditions, a desirable trait for real-world generalization. Figure 7D breaks down individual source-to-target transfer accuracy into two heatmaps, one for each model. While both methods perform well on the diagonal (source and target species being identical), off-diagonal entries reveal clearer differences. For example, CFM-GP maintains high $R^2$ when predicting from mouse to pig (0.7869) or rat to rabbit (0.8275), where CoupleVAE lags behind. In contrast, CoupleVAE shows slightly better performance from pig to rabbit and rat cases where the biological variance is relatively lower and may not require modeling dynamic transformation.

#### Relationship to phylogenetic divergence

To interpret cross-species performance variation in Fig. 7D, we quantified phylogenetic divergence between species using divergence times obtained from the TimeTree database [36]. Divergence times ranged from 11.6 MYA (mouse–rat) to 94 MYA (pairs involving pig; Supplementary Table S12). Across the six species pairs, mean cross-species performance (computed as the average bidirectional $R^2$ per species pair; Supplementary Table S12) showed only a weak negative association with divergence time (Spearman $\rho =-0.278$, $p=0.594$; Pearson $r=-0.195$, $p=0.712$). These results indicate that transfer accuracy does not degrade in a statistically monotonic manner with increasing evolutionary distance.

While closely related species (e.g. mouse–rat) exhibit strong transfer performance, several more evolutionarily distant species pairs also demonstrate high predictive accuracy. This suggests that cross-species generalization is influenced not solely by phylogenetic proximity but also by conserved regulatory programs and dataset-specific characteristics.

Importantly, these results indicate that CFM-GP not only learns perturbation directions aligned with species-specific gene expression but also preserves cross-species structure in the conditional flow space. While the advantages are modest in some directions, the general trend favors CFM-GP in more divergent or biologically complex settings. This capacity is particularly valuable for comparative genomics and preclinical translational studies, where human-like predictions must be extrapolated from non-human training domains.

### CFM-GP shows robust performance across varying experimental conditions and perturbations

#### Ablation study

##### Impact of MLP depth on model performance

To evaluate the sensitivity of CFM-GP to architectural complexity, we systematically varied the depth of the shared MLP [39, 40] from 1 to 10 layers while keeping all other components fixed. This ablation isolates the effect of nonlinear capacity on prediction accuracy, distributional alignment (MMD), and rank preservation (Spearman) (Supplementary Table S14). Our goal was to identify whether deeper networks improve modeling of complex gene perturbation patterns, or if excessive depth introduces overfitting or degradation.

Figure 7F presents the aggregate performance of CFM-GP across varying MLP depths [40], using a radar plot of $R^2$, Spearman correlation, and $1 - \mathrm{MMD}$. The baseline 3-layer configuration consistently achieves the highest scores across all three metrics, demonstrating an effective balance between accuracy and generalization. The 5-layer variant ranks second overall and performs best among the ablated configurations. Shallower networks (e.g. 1-layer) perform notably worse, while very deep networks (e.g. 10-layer) offer limited gains, likely due to overfitting. These results highlight medium-depth architectures, particularly the 3- and 5-layer models, as the most effective design choices.

Figure 7G illustrates the R² scores across varying MLP depths for each cell type. The baseline consistently achieves the highest *R*², as expected from the fully tuned model. Among ablated versions, the 5-layer model consistently recovers the most predictive accuracy across all cell types. Shallower (1-layer) and deeper (10-layer) configurations notably underperform, with the 1-layer variant exhibiting the steepest drop, especially in FCGR3A+ Mono and CD8T cells. These trends support baseline configuration as the most balanced depth for retaining predictive fidelity across cellular contexts.

Figure 7H tracks Spearman correlation across MLP depth variants for each cell type, providing insight into the model’s ability to preserve gene expression ordering. The baseline model achieves the highest rank fidelity overall, with the 5-layer variant again coming closest to recovering baseline performance. A single hidden layer substantially degrades correlation across all cell types, most notably in CD4T and B cells while moderate recovery is observed as depth increases to 2 and 5 layers. The 10-layer variant shows declining trends again, particularly in low-signal populations like CD14+Mono and FCGR3A+Mono, suggesting overparameterization may destabilize rank structure preservation.

Figure 7I presents MMD across varying MLP depths, offering a distributional perspective on how well predicted and real gene profiles align. The baseline model consistently yields the lowest MMD values, confirming its strong distributional calibration. Shallow variants (e.g. 1-layer) lead to notable increases in MMD for most cell types, especially CD14+Mono, CD8T, and NK indicating weaker distribution matching. Deeper configurations (layers 5 and 10) partially recover performance, but never match the original baseline. Interestingly, CD4T maintains near-zero MMD across all depths, suggesting either inherently easy-to-align distributions or metric saturation. Overall, the figure highlights that while moderate depth can stabilize performance, deeper networks risk diminishing returns or increased mismatch in more complex populations.

##### Importance of cell type conditioning for accurate perturbation modeling

We ablated the cell type conditioning component in the cross-attention and decoder modules to assess its role in guiding gene expression prediction. By training a variant of CFM-GP without explicit access to cell identity, we tested whether performance relies on cell type context to model heterogeneous responses shown in Supplementary Table S15. This experiment directly probes the model’s ability to generalize across cell types and uncovers the contribution of biological conditioning to multi-metric robustness.

Figure 7J illustrates the performance degradation across cell types when cell type conditioning is removed from CFM-GP. The most substantial impact is observed in the *R*^2^ metric (blue bars), where predictive accuracy drops by over 0.22 for several populations including CD4T, CD8T, and FCGR3A+Mono, highlighting the model’s strong reliance on cell type context to model accurate gene responses. Spearman rank correlation (orange bars) also suffers, with a maximum decline of approximately 0.16, especially for B and CD8T cells, indicating a weakened ability to preserve gene ordering. Interestingly, the $\Delta$MMD values (green bars) remain relatively low, suggesting that although expression ranking and magnitude suffer, the overall distributional alignment degrades less severely. This decoupling underscores that MMD alone may mask biologically important shifts in gene-specific behavior.

Figure 7K presents a ternary plot comparing the trade-offs among three key evaluation metrics *R*^2^, Spearman correlation, and $1{-}\mathrm{MMD}$ for CFM-GP models trained with (blue dots) and without (red squares) cell type conditioning. Each point represents a normalized performance profile for a specific cell type, where proximity to a vertex indicates stronger emphasis on that particular metric. The plot reveals a systematic shift in the red squares (ablated model) away from the *R*^2^ and Spearman corners and slightly toward the $1{-}\mathrm{MMD}$ vertex. This shift implies that removing cell type conditioning results in a notable degradation in predictive accuracy and rank preservation, consistent with the bar plot in Fig. 7J. At the same time, $1{-}\mathrm{MMD}$ remains relatively stable, possibly because it reflects global distributional alignment, which is less sensitive to fine-grained gene-wise structure. Interestingly, some red squares remain close to their baseline counterparts, suggesting a subset of cell types may be less dependent on explicit cell type features for general structure modeling. However, the consistent directional shift across most cell types highlights that cell type conditioning is critical for achieving a balanced optimization across all three axes, especially preserving nuanced gene relationships and ensuring biologically faithful predictions.

## Discussion

Understanding how cells respond to genetic or chemical perturbations across diverse contexts remains one of the central goals of functional genomics. While single-cell technologies have made it possible to directly measure transcriptional shifts at high resolution, their widespread use is limited by cost, scale, and experimental feasibility. Recent advances in perturbation modeling further highlight the diversity of methodological approaches in this space. GEARS [41] leverages graph-based architectures to model gene–gene interactions, while AttentionPert [42] introduces attention mechanisms to capture perturbation-specific regulatory effects. Similarly, scPRAM [43] emphasizes probabilistic regulatory activity modeling, and PerturBench (Wu *et al*., 2024, arXiv) provides a standardized benchmarking framework for perturbation prediction models [44][45][46]. In contrast to these approaches, CFM-GP formulates perturbation response as a continuous-time conditional flow, explicitly modeling gene expression transitions through a learned vector field conditioned on cell type. These perspectives are complementary, and future work may benefit from integrating graph priors, attention mechanisms, or standardized benchmarking protocols within continuous generative dynamics frameworks. Computational surrogates are therefore increasingly important, but existing approaches often require separate models for each cell type and struggle to generalize to unseen conditions. In this study, we proposed CFM-GP, a unified framework based on conditional flow matching, and demonstrated its ability to accurately model perturbation responses across multiple biological settings. Here, we reflect on the main findings, their implications, and the limitations of the study.

CFM-GP consistently achieved higher $R^2$ scores and Spearman correlations than state-of-the-art baselines across five datasets spanning infection, immune stimulation, and cancer. These results show that a single conditional model can learn perturbation dynamics that generalize across diverse cell types without needing separate models. Importantly, the gains were especially pronounced in the top differentially expressed genes, highlighting the method’s ability to capture biologically meaningful changes rather than global averages. From a practical perspective, this suggests that CFM-GP could reduce the need for costly experiments by providing reliable in silico predictions across under-sampled contexts.

Beyond predictive accuracy, we assessed whether CFM-GP preserved the global structure of cell populations. UMAP visualizations and MMD scores demonstrated that predicted perturbation profiles maintained both intra-cell-type coherence and inter-cell-type boundaries, closely mirroring ground-truth distributions. This is critical because perturbation effects often manifest not only in mean shifts of gene expression but also in the redistribution of cells within and across states. By capturing these patterns, CFM-GP provides a more faithful representation of population-level responses, a property that is essential for downstream applications such as clustering, trajectory inference, or identification of resistant subpopulations.

A major strength of CFM-GP lies in its ability to recover biologically relevant pathways from predicted profiles. Enrichment analyses confirmed that the model not only reproduced overall gene expression changes but also preserved the functional signatures of perturbations, such as immune signaling cascades or drug-induced transcriptional programs. This interpretability is essential for translational applications, as models must go beyond statistical fit to provide mechanistic insight. By retaining pathway-level information, CFM-GP may serve as a powerful hypothesis generator for functional genomics and therapeutic discovery. Perhaps the most demanding test involved cross-species prediction. The ability of CFM-GP to transfer perturbation knowledge across species underscores its potential for bridging experimental systems with human biology. This opens the possibility of leveraging well-characterized model organisms to predict responses in less accessible contexts, thereby accelerating translational research. While the performance drop relative to within-species predictions indicates room for improvement, the fact that cross-species generalization is feasible at all highlights the flexibility of the conditional flow-matching framework.

Together, these results suggest that CFM-GP offers a scalable and generalizable solution for perturbation-response modeling. By unifying diverse cell types and perturbations within a single model, it provides efficiency gains and reduces fragmentation in computational approaches. Practically, this could facilitate virtual perturbation screens, guide experimental design by prioritizing conditions of interest, and accelerate drug target identification by predicting how interventions manifest across cellular contexts. As single-cell datasets continue to grow in size and diversity, approaches like CFM-GP will be crucial for turning raw measurements into predictive, actionable knowledge.

Several limitations must be acknowledged. First, although CFM-GP generalizes across datasets, its performance remains dependent on the diversity and quality of available training data. Rare cell types and sparsely measured perturbations remain challenging. Second, while we benchmarked extensively on real datasets, we did not include synthetic datasets with ground truth, which would provide an orthogonal validation of model calibration. Third, the current implementation is focused on transcriptomic data; extending the framework to multi-omic settings, such as chromatin accessibility or proteomics, could further expand its utility. Finally, computational cost may become a bottleneck for atlas-scale applications, motivating future work on model compression and efficient inference.

The results establish CFM-GP as a robust and interpretable framework for perturbation prediction, while also highlighting areas for continued improvement. By balancing accuracy, distributional fidelity, and biological interpretability, CFM-GP contributes to the growing toolkit of computational methods that aim to make perturbation-response modeling scalable, reproducible, and ultimately more accessible to the broader genomics community.

## Conclusions

In summary, our findings demonstrate that CFM-GP provides a principled and effective solution for modeling perturbation responses in single-cell transcriptomics. By framing perturbation dynamics as time-dependent vector fields, the method enables biologically grounded transformations from control to perturbed states. Across five diverse datasets and a cross-species transfer setting, CFM-GP consistently outperformed state-of-the-art baselines in predictive accuracy, distributional alignment, and pathway recovery.

Looking forward, CFM-GP establishes a foundation for generalizable perturbation modeling with direct implications for functional genomics and drug discovery. Future extensions will focus on systematic evaluation of novel perturbations, integration of structural and regulatory priors, and incorporation of uncertainty quantification. Taken together, these results position CFM-GP as a significant methodological advance toward scalable, biologically faithful modeling of cellular responses.

## Supplementary Material

lqag087_Supplemental_File

## Data Availability

All datasets used in this study are publicly available: COVID-19 [29]: GEO accession GSE145926 PBMC (IFN-$\beta$ stimulation) [44]: GEO accession GSE96583 Glioblastoma (Panobinostat treatment) [45]: GEO accession GSE148842 Lupus (IFN-$\beta$ stimulation) [44]: GEO accession GSE96583 Statefate (cytokine–environment interactions) [46]: GEO accession GSE140802 Cross-species dataset: UK Biostudies accession E-MTAB-6754

## Code availability

CFM-GP is implemented in Python using standard deep learning and bioinformatics libraries. The source code, including training, evaluation, and figure generation scripts, is freely available at https://github.com/abrarrahmanabir/CFM-GP.git and https://doi.org/10.7294/32086827.

## References

[1] Ja D, Charpentier E. Genome editing. The new frontier of genome engineering with CRISPR–Cas9. Science. 2014;346:1258096. 10.1126/science.1258096. 25430774

[2] Wang L, Zhou L, Li M et al. Genome-wide CRISPR/Cas9 knockout screening uncovers ZNF319 as a novel tumor suppressor critical for breast cancer metastasis. Biochem Biophys Res Commun. 2022;589:107–15. 10.1016/j.bbrc.2021.12.023. 34902746

[3] Conte Jr D, MacNeil LT, Walhout AJ et al. RNA interference in *Caenorhabditis elegans*. Curr Protoc Mol Biol. 2015;109:26–3. 10.1002/0471142727.mb2603s109. PMC5396541 25559107

[4] Fire A, Xu S, Montgomery MK et al. Potent and specific genetic interference by double-stranded RNA in Caenorhabditis elegans. Nature. 1998;391:806–11. 10.1038/35888. 9486653

[5] Szalai B, Veres DV. Application of perturbation gene expression profiles in drug discovery—from mechanism of action to quantitative modelling. Front Syst Biol. 2023;3:1126044. 10.3389/fsysb.2023.1126044. 40809500 PMC12341996

[6] Dixit A, Parnas O, Li B et al. Perturb-Seq: dissecting molecular circuits with scalable single-cell RNA profiling of pooled genetic screens. Cell. 2016;167:1853–66. 10.1016/j.cell.2016.11.038. 27984732 PMC5181115

[7] Ishikawa M, Sugino S, Masuda Y et al. RENGE infers gene regulatory networks using time-series single-cell RNA-seq data with CRISPR perturbations. Commun Biol. 2023;6:1290. 10.1038/s42003-023-05594-4. 38155269 PMC10754834

[8] Park BS, Lee M, Kim J et al. Perturbomics: CRISPR–Cas screening-based functional genomics approach for drug target discovery. Exp Mol Med. 2025;57:1443–54. 40588529 10.1038/s12276-025-01487-0 PMC12322284

[9] Datlinger P, Rendeiro AF, Schmidl C et al. Pooled CRISPR screening with single-cell transcriptome readout. Nat Methods. 2017;14:297–301. 10.1038/nmeth.4177. 28099430 PMC5334791

[10] Peidli S, Green TD, Shen C et al. scperturb: harmonized single-cell perturbation data. Nature Methods. 2024;21:531–40. 38279009 10.1038/s41592-023-02144-y PMC12220817

[11] Märtens K, Donovan-Maiye R, Ferkinghoff-Borg J. Enhancing generative perturbation models with llm-informed gene embeddings. In: ICLR 2024 Workshop on Machine Learning for Genomics Explorations. Vienna, Austria, 2024.

[12] Dip SA, Mallick D, Acharjee Shuvo U et al. Large language model agents for biological intelligence across genomics, proteomics, spatial biology, and biomedicine. Brief Bioinform. 2026;27:bbag110. 10.1093/bib/bbag110. 41883029 PMC13017847

[13] Gavriilidis GI, Vasileiou V, Orfanou A et al. A mini-review on perturbation modelling across single-cell omic modalities. Computat Struct Biotechnol J. 2024;23:1886. 10.1016/j.csbj.2024.04.058. PMC11076269 38721585

[14] Norman TM, Horlbeck MA, Replogle JM et al. Exploring genetic interaction manifolds constructed from rich single-cell phenotypes. Science. 2019;365:786–93. 10.1126/science.aax4438. 31395745 PMC6746554

[15] Srivatsan SR, McFaline-Figueroa JL, Ramani V et al. Massively multiplex chemical transcriptomics at single-cell resolution. Science. 2020;367:45–51. 10.1126/science.aax6234. 31806696 PMC7289078

[16] Replogle JM, Norman TM, Xu A et al. Combinatorial single-cell CRISPR screens by direct guide RNA capture and targeted sequencing. Nat Biotechnol. 2020;38:954–61. 10.1038/s41587-020-0470-y. 32231336 PMC7416462

[17] Lotfollahi M, Wolf FA, Theis FJ. scGen predicts single-cell perturbation responses. Nat Methods. 2019;16:715–21. 10.1038/s41592-019-0494-8. 31363220

[18] Lotfollahi M, Naghipourfar M, Theis FJ et al. Conditional out-of-distribution generation for unpaired data using transfer VAE. Bioinformatics. 2020;36:i610–7. 10.1093/bioinformatics/btaa800. 33381839

[19] Ahlmann-Eltze C, Huber W, Anders S. Deep-learning-based gene perturbation effect prediction does not yet outperform simple linear baselines. Nat Methods. 2025;22:1657–61. 40759747 10.1038/s41592-025-02772-6 PMC12328236

[20] Sohn K, Lee H, Yan X. Learning structured output representation using deep conditional generative models. Adv Neur Inf Proc Syst. 2015;28.

[21] Nicol PB, Paulson D, Qian G et al. Robust identification of perturbed cell types in single-cell RNA-seq data. Nat Commun. 2024;15:7610. 10.1038/s41467-024-51649-3. 39218971 PMC11366752

[22] Wei X, Dong J, Wang F. scPreGAN, a deep generative model for predicting the response of single-cell expression to perturbation. Bioinformatics. 2022;38:3377–84. 10.1093/bioinformatics/btac357. 35639705

[23] Wu Y, Liu J, Xiao Y et al. CoupleVAE: coupled variational autoencoders for predicting perturbational single-cell RNA sequencing data. Brief Bioinform. 2025;26:bbaf126 10.1093/bib/bbaf126. 40178283 PMC11966612

[24] Lotfollahi M, Klimovskaia Susmelj A, De Donno C et al. Predicting cellular responses to complex perturbations in high-throughput screens. Mol Syst Biol. 2023;19:e11517. 10.15252/msb.202211517. 37154091 PMC10258562

[25] Bunne C, Stark SG, Gut G et al. Learning single-cell perturbation responses using neural optimal transport. Nat Methods. 2023;20:1759–68. 10.1038/s41592-023-01969-x. 37770709 PMC10630137

[26] Klein D, Fleck JS, Bobrovskiy D et al. CellFlow enables generative single-cell phenotype modeling with flow matching. bioRxiv. 2025;2025–04.

[27] Bereket M, Karaletsos T. Modelling cellular perturbations with the sparse additive mechanism shift variational autoencoder. Adv Neur Inf Proc Syst. 2023;36:1–12.

[28] Palma A, Theis FJ, Lotfollahi M. Predicting cell morphological responses to perturbations using generative modeling. Nat Commun. 2025;16:505. 10.1038/s41467-024-55707-8. 39779675 PMC11711326

[29] Lotfollahi M, Naghipourfar M, Luecken MD et al. Mapping single-cell data to reference atlases by transfer learning. Nat Biotechnol. 2022;40:121–30. 10.1038/s41587-021-01001-7. 34462589 PMC8763644

[30] Cameron AC, Windmeijer FA. An R-squared measure of goodness of fit for some common nonlinear regression models. J Econometrics. 1997;77:329–42. 10.1016/S0304-4076(96)01818-0.

[31] Gretton A, Borgwardt KM, Rasch MJ et al. A kernel two-sample test. J Mach Learn Res. 2012;13:723–73.

[32] Spearman C . The proof and measurement of association between two things. Am J Psychol. 1987;100:441–71. 10.2307/1422689. 3322052

[33] Subramanian A, Tamayo P, Mootha VK et al. Gene set enrichment analysis: a knowledge-based approach for interpreting genome-wide expression profiles. Proc Natl Acad Sci. 2005;102:15545–50. 10.1073/pnas.0506580102. 16199517 PMC1239896

[34] Nair V, Hinton GE. Rectified linear units improve restricted boltzmann machines. In: Proceedings of the 27th international conference on machine learning (ICML-10), pp. 807–14. Haifa, Israel, 2010,

[35] Kingma DP, Ba J. Adam: a method for stochastic optimization. In: Proceedings of the International Conference on Learning Representations (ICLR). San Diego, CA, USA, 2015.

[36] Kumar S, Stecher G, Suleski M et al. TimeTree: a resource for timelines, timetrees, and divergence times. Mol Biol Evol. 2017;34:1812–9. 10.1093/molbev/msx116. 28387841

[37] McInnes L, Healy J, Saul N et al. UMAP: Uniform Manifold Approximation and Projection. J Open Source Softw. 2018;3:861. 10.21105/joss.00861.

[38] Becht E, McInnes L, Healy J et al. Dimensionality reduction for visualizing single-cell data using UMAP. Nat Biotechnol. 2019;37:38–44. 10.1038/nbt.4314. 30531897

[39] Rosenblatt F . The perceptron: a probabilistic model for information storage and organization in the brain. Psychol Rev. 1958;65:386. 10.1037/h0042519. 13602029

[40] Rumelhart DE, Hinton GE, Williams RJ. Learning representations by back-propagating errors. Nature. 1986;323:533–6. 10.1038/323533a0.

[41] Roohani Y, Huang K, Leskovec J. Predicting transcriptional outcomes of novel multigene perturbations with GEARS. Nat Biotechnol. 2024;42:927–35. 10.1038/s41587-023-01905-6. 37592036 PMC11180609

[42] Bai D, Ellington CN, Mo S et al. AttentionPert: accurately modeling multiplexed genetic perturbations with multi-scale effects. Bioinformatics. 2024;40:i453–61. 10.1093/bioinformatics/btae244. 38940174 PMC11211811

[43] Jiang Q, Chen S, Chen X et al. scPRAM accurately predicts single-cell gene expression perturbation response based on attention mechanism. Bioinformatics. 2024;40:btae265. 10.1093/bioinformatics/btae265. 38625746 PMC11076148

[44] Kang HM, Subramaniam M, Targ S et al. Multiplexed droplet single-cell RNA-sequencing using natural genetic variation. Nat Biotechnol. 2018;36:89–94. 10.1038/nbt.4042. 29227470 PMC5784859

[45] Zhao W, Dovas A, Spinazzi EF et al. Deconvolution of cell type-specific drug responses in human tumor tissue with single-cell RNA-seq. Genome Med. 2021;13:82. 10.1186/s13073-021-00894-y. 33975634 PMC8114529

[46] Weinreb C, Rodriguez-Fraticelli A, Camargo FD et al. Lineage tracing on transcriptional landscapes links state to fate during differentiation. Science. 2020;367:eaaw3381. 10.1126/science.aaw3381. 31974159 PMC7608074

