# Supplementary material for: CFM-GP: unified conditional flow matching to learn gene perturbation across cell types
Source: NAR Genom Bioinform. 2026 Aug 10;8(3):lqag087. doi: 10.1093/nargab/lqag087 (PMC13454845; doi:10.1093/nargab/lqag087)
Supplement: Supplementary file 1 [file lqag087_supplemental_file.pdf]

Supplementary Information for
CFM-GP: Unified Conditional Flow Matching to Learn Gene Perturbation Across Cell Types

Abrar Rahman Abir, Sajib Acharjee Dip, and Liqing Zhang

1 Supplementary Note 1. Datasets and Preprocessing

1.1 Datasets Overview

COVID-19 Peripheral Airway Dataset

We used a publicly available single-cell RNA-seq dataset profiling the peripheral airway epithelium and immune cells from COVID-19 patients and healthy controls [1], available under accession code GSE145926. The dataset contains 4713 training, 602 validation, and 556 test cells, each with 6000 genes. Six immune and epithelial cell types are represented: CD8 T cells, macrophages, NK cells, neutrophils, secretory cells, and myeloid dendritic cells (mDCs). Each split includes paired control and perturbed profiles per cell, enabling direct modeling of transcriptional shifts. Macrophages and neutrophils are the most abundant cell types, accounting for the majority of paired samples across all data splits.

PBMC Stimulation Dataset

We also used single-cell RNA-seq dataset of peripheral blood mononuclear cells (PBMCs) stimulated ex vivo, available under accession code GSE96583. The dataset contains 5998 training, 765 validation, and 2924 test cells, each with 6998 genes. It covers seven major immune cell types: B cells, CD4 and CD8 T cells, CD14^{+} monocytes, FCGR3A^{+} monocytes, dendritic cells, and NK cells. Each cell has paired control and stimulated profiles, enabling precise modeling of perturbation effects. CD4 T cells are the most abundant, followed by monocytes and B cells, offering diverse signal across innate and adaptive immune compartments.

Glioblastoma Drug Response Dataset

To evaluate perturbation prediction in a cancer context, we utilized a single-cell RNA-seq dataset profiling glioblastoma patient-derived cells under drug treatment [2], available under accession code GSE148842. The dataset includes 30,842 cells with 1000 genes, derived from six unique patient samples (PW029, PW030, PW032, PW034, PW036, and PW052), which we treat as distinct pseudo–cell types. Paired control and treated profiles are provided for each sample, with PW034 and PW036 comprising the largest subsets (15,288 and 6120 cells, respectively). The data were split into training (21,589 cells), validation (4626 cells), and test (4627 cells) sets, enabling evaluation of generalization across patients.

Systemic Lupus Erythematosus (SLE) Dataset

We analyzed a peripheral blood single-cell RNA-seq dataset from lupus patients [3], sourced from GSE96583, comprising 13,795 cells and 1000 genes. The dataset spans eight immune cell types, including CD4 and CD8 T cells, B cells, monocyte subsets, dendritic cells, NK cells, and megakaryocytes. We used a 70:15:15 split across training, validation, and test sets. CD4 T cells and CD14^{+} monocytes were the most abundant, offering a rich basis for modeling diverse immune perturbation responses.

Cytokine-Driven Progenitor Fate Dataset (Statefate)

This dataset [4], obtained from GSE140802, captures cytokine-induced transcriptional changes in hematopoietic progenitor cells. It includes 28,249 cells across two sorted populations: Lin-Kit^{+}Sca1^{+} and Lin-Kit^{+}Sca1^{-}. The data are split into training (19,774), validation (4237), and test (4238) sets, with over

20,000 paired control and perturbed cells in the Sca1$^+$ group alone—making it well-suited for modeling lineage-specific perturbation effects.

1.2 Dataset Composition and Perturbation Balance

All datasets used in this study consist of strictly paired control and perturbed single-cell expression profiles. For every control cell included in training, validation, or testing, a corresponding perturbed profile of the same cell type is available. Consequently, the proportion of perturbed cells is exactly 50% in every dataset split. Differences in UMAP separation (Figure 2) therefore reflect biological perturbation magnitude rather than imbalance in perturbation proportion.

To ensure transparency, we report detailed dataset composition statistics below, including the number of paired samples per split and per cell type.

COVID-19 Peripheral Airway Dataset (GSE145926)

Table S1: COVID-19 dataset composition across splits. Each split contains paired control and perturbed cells (1:1 ratio).

Split	Total Paired Cells	Control Cells	Perturbed Cells
Train	4713	4713	4713
Validation	602	602	602
Test	556	556	556

Table S2: COVID-19 training set composition per cell type.

Cell Type	Paired Cells
Macrophages	2159
Neutrophil	1283
NK	534
Secretory	308
mDC	268
CD8 T	161

Cell-Type Distribution (Training Set)

PBMC IFN-β Stimulation Dataset (GSE96583)

Table S3: PBMC dataset composition across splits (1:1 control–perturbed pairing).

Split	Total Paired Cells	Control Cells	Perturbed Cells
Train	5998	5998	5998
Validation	765	765	765
Test	2924	2924	2924

Table S4: PBMC training set composition per cell type.

Cell Type	Paired Cells
CD4T	2437
FCGR3A+ Mono	967
B	707
CD14+ Mono	531
CD8T	487
NK	462
Dendritic	407

Cell-Type Distribution (Training Set)

Glioblastoma Drug Response Dataset (GSE148842)

Table S5: Glioblastoma dataset composition (paired treated vs. untreated cells).

Total Paired Cells	30,842
Training Cells	21,589
Validation Cells	4,626
Test Cells	4,627

Table S6: Glioblastoma paired cells per patient sample.

Sample ID	Paired Cells
PW034	15,288
PW036	6,120
PW030	3,942
PW032	2,508
PW029	1,696
PW052	1,288

Sample Distribution

Systemic Lupus Erythematosus Dataset (GSE96583)

Table S7: Lupus dataset composition (1:1 control–perturbed pairing).

Total Paired Cells	13,795
Training Cells (70%)	9,656
Validation Cells (15%)	2,069
Test Cells (15%)	2,070

Table S8: Lupus dataset paired cells per immune cell type.

Cell Type	Paired Cells
CD4 T cells	5,889
CD14+ Monocytes	2,990
B cells	1,350
CD8 T cells	1,205
NK cells	1,071
FCGR3A+ Monocytes	926
Dendritic cells	232
Megakaryocytes	132

Cell-Type Distribution

Statefate Cytokine-Driven Progenitor Dataset (GSE140802)

Table S9: Statefate dataset composition (paired control–perturbed profiles).

Total Paired Cells	28,249
Training Cells	19,774
Validation Cells	4,237
Test Cells	4,238

Table S10: Statefate paired cells per progenitor population.

Population	Paired Cells
Lin-Kit+Sca1+	20,456
Lin-Kit+Sca1−	7,793

1.3 Preprocessing and Splits

For each of the five datasets described in the main text (COVID-19, PBMC IFN-β, Glioblastoma drug treatment, Lupus IFN-β, and Statefate cytokine stimulation), we constructed paired control–perturbation profiles with consistent gene ordering across conditions. Each split was serialized into PyTorch `.pt` files to ensure efficient loading during training and evaluation. Every file contains:

- `x_ctrl` ($N \times G$): control expression profiles,
- `x_pert` ($N \times G$): matched perturbed profiles,
- `cell_type` (N): integer indices denoting the cell type for each observation,
- `cell_type_mapping`: dictionary mapping cell type labels to indices,
- `gene_names`: list of gene identifiers (optional, used in ranking analyses).

Upstream preprocessing (normalization, log transformation, highly variable gene (HVG) selection, and batch harmonization where applicable) was performed before tensorization and is identical to the procedures described in Supplementary Note 1.

We maintained three disjoint splits for every dataset:

1. **Training set**, used to fit model parameters,
2. **Validation set**, used exclusively for hyperparameter tuning and model checkpoint selection,
3. **Test set**, held out completely until final evaluation.

Splitting was stratified by cell type to maintain proportional representation across subsets. Where donor or patient identifiers were available, we enforced donor disjointness to prevent information leakage.

2 Supplementary Note 2. Model Training and Inference

2.1 Training Details

All models were trained using the Conditional Flow Matching objective described in the main text. Unless otherwise stated, training was performed on NVIDIA GPUs with mixed precision enabled.

Architecture. The conditional flow model consisted of a multilayer perceptron (MLP) operating on the concatenation of the interpolated state $x(t)$, the original control state x_c, a learned 16-dimensional cell type embedding, and a 16-dimensional time embedding obtained by applying a linear transformation followed by ReLU to the scalar t. The backbone network had two hidden layers of width 256 with ReLU activations and produced a G-dimensional velocity vector, where G is the number of genes retained after preprocessing.

Optimization. We used the Adam optimizer with a base learning rate of 1×10^{-3} and default momentum parameters ($\beta_1 = 0.9,\ \beta_2 = 0.999$). Models were trained for 50. A batch size of 32 was used throughout.

Validation and checkpointing. At the end of each epoch, models were evaluated on the validation set using the same loss function. The checkpoint with the lowest validation loss was retained for all downstream evaluation. Training and validation losses were logged at every epoch to ensure stability and reproducibility.

Reproducibility. Random seeds were fixed for PyTorch and NumPy during training runs to minimize stochastic variation. All hyperparameters and configuration files are available in the accompanying code repository.

Training Time We report the mean training time of 10 runs in Table S11 CFM-GP is by far the fastest method while simultaneously achieving state-of-the-art performance. This efficiency arises naturally from the design of CFM-GP, which requires training only a single model to generalize across all cell types. In contrast, competing methods such as scDist, scPreGAN, trVAE, scGen, CVAE, and CoupleVAE must train separate models for each cell type, leading to substantially higher computational costs. As a result, CFM-GP not only reduces runtime compared to existing approaches but also provides a more scalable solution for diverse datasets.

Table S11: Training time (in seconds) of different methods across datasets.

Dataset	CFM-GP	scDist	scPreGAN	trVAE	scGen	CVAE	CoupleVAE
COVID-19	46.5	1404.8	16314.4	745.2	9348.1	1600.8	276.5
PBMC	60.0	2137.8	24826.2	1134.0	14225.4	2436.0	420.1
Glioblastoma	140.1	4275.6	49652.4	2268.0	28450.8	4872.0	840.9
Lupus	50.3	2036.0	23644.0	1080.0	13548.0	2320.0	400.4
Statefate	130.7	1323.4	15368.6	702.0	8806.2	1508.0	260.3

2.2 Inference procedure

To generate predicted perturbation profiles from a trained Conditional Flow Matching model, we integrated the learned conditional vector field from $t = 0$ (control) to $t = 1$ (perturbation). Integration was performed deterministically using a uniform 10-step Euler scheme:

$$x^{(0)} = x_c, \quad x^{(k+1)} = x^{(k)} + \Delta t \cdot v_\theta(x^{(k)}, t_k \mid x_c, c), \quad t_k = \tfrac{k}{9},\ \Delta t = 0.1.$$

Here, x_c is the control expression profile, c is the cell type index, and v_θ is the neural network approximating the velocity field. After 10 iterations, the final state $x^{(10)}$ was taken as the predicted perturbed profile $\hat{y}$.

2.3 Evaluation metrics

We report four complementary metrics, each computed independently per cell type in the test set.

Coefficient of determination ($\mathbf{R}^2$). We defined R^2 as the squared Pearson correlation between the mean predicted profile $\overline{\hat{y}}$ and the mean ground-truth perturbed profile $\overline{y}$ across all genes. This provides a global measure of variance explained at the cell-type level. To assess performance on informative features, we also computed R^2 restricted to the top 100 differentially expressed genes (DEGs). DEGs were identified by comparing *real perturbed* versus *control* cells using the Wilcoxon rank-sum test implemented in `scanpy.tl.rank_genes_groups`. Genes were ranked by absolute test statistic, and the top 100 were used to compute R^2 between $\overline{\hat{y}}$ and $\overline{y}$ on this subset.

Distributional similarity (MMD). We quantified how well predicted and real perturbed distributions aligned using the Maximum Mean Discrepancy (MMD). For each cell type, we estimated the squared MMD between predicted cells $\{\hat{y}_i\}$ and true perturbed cells $\{y_i\}$ under a Gaussian (RBF) kernel:

$$\mathrm{MMD}^2 = \frac{1}{n^2}\sum_{i,j} k(\hat{y}_i, \hat{y}_j) + \frac{1}{m^2}\sum_{i,j} k(y_i, y_j) - \frac{2}{nm}\sum_{i,j} k(\hat{y}_i, y_j).$$

Kernel bandwidth γ was set by the median heuristic on the pooled data $\{\hat{y}\} \cup \{y\}$.

Spearman rank correlation. To assess preservation of gene-level ordering, we computed Spearman's rank correlation coefficient (ρ) between mean predicted expression and mean real expression across genes in each cell type. This highlights consistency in relative up- and down-regulation independent of scale.

Pathway enrichment overlap. We performed preranked Gene Set Enrichment Analysis (GSEA) on gene-wise differential expression scores ($\hat{y} - x_c$ for predictions and $y - x_c$ for real data). Pathways from the Hallmark and KEGG collections were ranked by normalized enrichment score (NES). Overlap was quantified by the Jaccard index between the top-10 pathways recovered from predicted and real perturbations.

2.4 Output

All metrics are reported per cell type, with full tables provided in Supplementary Table S13. Summary statistics (means, standard deviations, and representative figures) are included in the main text. CSV outputs from the evaluation pipeline, including R^2, MMD, and Spearman scores, are deposited with the code repository to ensure reproducibility.

Supplementary Tables

Table S12: Phylogenetic divergence times from TimeTree [5] and cross-species performance from Fig. 7d. Mean R^2 is computed as the average of bidirectional transfers between each species pair.

Species pair	Divergence (MYA)	$R^2_{A\to B}$	$R^2_{B\to A}$	Mean R^2
Mouse–Rat	11.6	0.844	0.802	0.823
Mouse–Rabbit	79	0.783	0.777	0.780
Rat–Rabbit	79	0.828	0.854	0.841
Mouse–Pig	94	0.787	0.761	0.774
Rat–Pig	94	0.854	0.827	0.841
Rabbit–Pig	94	0.813	0.809	0.811

Table S13: Key Hyperparameters used to conduct the experiments

**Hyperparameter**	**Value/Default**	**Description**
Data / Loader		
`train_path`	(user-specified)	Path to training dataset (.pt file)
`val_path`	(user-specified)	Path to validation dataset (.pt file)
`test_path`	(user-specified)	Path to test dataset (.pt file)
`batch_size`	32	Mini-batch size for DataLoader
Model Architecture		
`input_dim`	(auto-inferred)	Number of gene features per cell
`num_cell_types`	(auto-inferred)	Number of distinct cell types
`hidden_dim`	256	Width of hidden layers in MLP
`cell_embedding_dim`	16	Embedding size for cell type
`time_embedding_dim`	16	Embedding size for time t
`num_mlp_layers`	3	Number of linear layers (excluding embeddings)
Training		
`epoch`	50 (default)	Number of training epochs
`lr`	1×10^{-4}	Adam learning rate
`optimizer`	Adam	Optimizer used
`loss_fn`	MSELoss	Mean squared error loss function
`device`	cuda/cpu	Device for training
`save_path`	model.pt	Path to save the trained model
Evaluation		
`n_steps`	10	Steps for iterative trajectory integration
`out_prefix`	CFM	Prefix for evaluation result files
`save_dir`	./results/	Directory for evaluation output
`kernel`	rbf	MMD kernel (for metric evaluation)
`gamma` (MMD)	1.0	RBF kernel gamma for MMD computation
`top_100_DEGs`	100	Top DEGs for cell-type specific R^2 evaluation

Table S14: Effect of varying MLP depth in CFM-GP. We report performance across seven PBMC cell types using R^2, MMD, and Spearman correlation. The baseline model uses the default MLP configuration (3 layers). Comparisons are made with 1, 2, 5, and 10-layer MLP variants.

**Cell Type**	**Baseline**			**1 Layer**			**2 Layers**			**5 Layers**			**10 Layers**		
	R^2	MMD	Spearman	R^2	MMD	Spearman	R^2	MMD	Spearman	R^2	MMD	Spearman	R^2	MMD	Spearman
B	0.9903	0.0182	0.760	0.8590	0.0210	0.6582	0.9028	0.0193	0.7113	0.9403	0.0188	0.7536	0.8626	0.0211	0.6605
CD14+Mono	0.9955	0.0241	0.584	0.8635	0.0280	0.5052	0.9045	0.0255	0.5473	0.9457	0.0249	0.5790	0.8668	0.0281	0.5072
CD4T	0.9696	0.0008	0.879	0.8327	0.0009	0.7716	0.8740	0.0008	0.8267	0.9210	0.0009	0.8720	0.8366	0.0009	0.7738
CD8T	0.9537	0.0377	0.678	0.8201	0.0443	0.5940	0.8642	0.0416	0.6378	0.9045	0.0394	0.6690	0.8226	0.0446	0.5965
Dendritic	0.9870	0.0364	0.644	0.8581	0.0428	0.5631	0.9026	0.0399	0.6059	0.9356	0.0380	0.6340	0.8615	0.0432	0.5649
FCGR3A+Mono	0.9457	0.0152	0.628	0.8037	0.0179	0.5482	0.8621	0.0163	0.5914	0.9005	0.0158	0.6172	0.8083	0.0180	0.5505
NK	0.9739	0.0370	0.686	0.8418	0.0447	0.5873	0.8893	0.0412	0.6454	0.9245	0.0389	0.6717	0.8444	0.0450	0.5899

Table S15: Ablation analysis of cell type conditioning in CFM-GP. Performance is reported across seven PBMC cell types with and without cell type conditioning. Metrics include coefficient of determination (R^2), Maximum Mean Discrepancy (MMD), and Spearman rank correlation.

**Cell Type**	**Baseline (w/ Cell Type)**			**Without Cell Type**		
	R^2	MMD	Spearman	R^2	MMD	Spearman
B	0.9903	0.0182	0.760	0.8205	0.0310	0.6026
CD14+Mono	0.9955	0.0241	0.584	0.7708	0.0478	0.4874
CD4T	0.9696	0.0008	0.879	0.7441	0.0009	0.7518
CD8T	0.9537	0.0377	0.678	0.7153	0.0637	0.5214
Dendritic	0.9870	0.0364	0.644	0.8163	0.0425	0.5536
FCGR3A+Mono	0.9457	0.0152	0.628	0.6949	0.0180	0.5251
NK	0.9739	0.0370	0.686	0.8151	0.0424	0.5639

Table S16: R^2 predictive accuracy across PBMC cell types for All Genes and Top 100 DEGs. Rows represent models; columns indicate cell types. Percentage improvements for CFM-GP over CoupleVAE are shown in the final rows.

**Model (Split)**	**FCGR3A+Mono**	**CD4T**	**B**	**CD8T**	**CD14+Mono**	**NK**	**Dendritic**
scDist (All Genes)	0.86	0.97	0.79	0.63	0.79	0.61	0.72
scPreGAN (All Genes)	0.90	0.92	0.91	0.84	0.87	0.85	0.85
trVAE (All Genes)	0.73	0.90	0.86	0.92	0.90	0.77	0.86
scGen (All Genes)	0.66	0.94	0.96	0.82	0.58	0.94	0.66
CVAE (All Genes)	0.63	0.83	0.78	0.78	0.58	0.74	0.64
CoupleVAE (All Genes)	0.98	0.99	0.96	0.94	0.98	0.93	0.96
**CFM-GP (All Genes)**	**0.9903**	**0.9955**	**0.9696**	**0.9537**	**0.9870**	**0.9457**	**0.9739**
scDist (Top 100 DEGs)	0.95	0.97	0.92	0.85	0.91	0.82	0.90
scPreGAN (Top 100 DEGs)	0.77	0.90	0.88	0.87	0.79	0.86	0.75
trVAE (Top 100 DEGs)	0.70	0.96	0.90	0.93	0.83	0.74	0.84
scGen (Top 100 DEGs)	0.41	0.98	0.96	0.74	0.29	0.98	0.36
CVAE (Top 100 DEGs)	0.40	0.65	0.59	0.64	0.30	0.64	0.35
CoupleVAE (Top 100 DEGs)	0.99	0.99	0.98	0.95	0.98	0.97	0.97
**CFM-GP (Top 100 DEGs)**	**0.9982**	**0.9998**	**0.9941**	**0.9946**	**0.9954**	**0.9758**	**0.9966**
% Improvement (All Genes)	+1.05%	+0.55%	+1.00%	+1.46%	+0.71%	+1.69%	+1.45%
% Improvement (Top 100 DEGs)	+0.83%	+0.99%	+1.43%	+4.71%	+1.57%	+0.60%	+2.74%

Table S17: R^2 predictive accuracy across glioblastoma samples for All Genes and Top 100 DEGs. Rows represent models; columns indicate sample IDs. Percentage improvements for CFM-GP over CoupleVAE are shown in the final rows.

**Model (Split)**	**PW029**	**PW030**	**PW032**	**PW034**	**PW036**	**PW052**
CoupleVAE (All Genes)	0.962	0.966	0.943	0.963	0.970	0.945
scPreGAN (All Genes)	0.953	0.948	0.935	0.958	0.962	0.949
trVAE (All Genes)	0.948	0.967	0.927	0.941	0.966	0.949
scDist (All Genes)	0.928	0.933	0.926	0.945	0.963	0.919
scGen (All Genes)	0.932	0.942	0.931	0.924	0.928	0.909
CVAE (All Genes)	0.936	0.939	0.901	0.937	0.935	0.930
**CFM-GP (All Genes)**	**0.978**	**0.983**	**0.960**	**0.985**	**0.986**	**0.970**
CoupleVAE (Top 100 DEGs)	0.957	0.939	0.926	0.904	0.954	0.948
scPreGAN (Top 100 DEGs)	0.870	0.941	0.942	0.891	0.914	0.930
trVAE (Top 100 DEGs)	0.921	0.857	0.915	0.966	0.860	0.853
scDist (Top 100 DEGs)	0.946	0.927	0.942	0.840	0.861	0.822
scGen (Top 100 DEGs)	0.781	0.847	0.827	0.843	0.871	0.900
CVAE (Top 100 DEGs)	0.788	0.798	0.866	0.762	0.867	0.917
**CFM-GP (Top 100 DEGs)**	**0.988**	**0.996**	**0.992**	**0.989**	**0.989**	**0.991**
% Improvement (All Genes)	+1.66	+1.79	+1.76	+2.31	+1.59	+2.66
% Improvement (Top 100 DEGs)	+3.23	+6.04	+7.11	+9.36	+3.68	+4.58

Table S18: R^2 predictive accuracy across lupus PBMC cell types for All Genes and Top 100 DEGs. Rows represent models; columns indicate cell types. Percentage improvements for CFM-GP over CoupleVAE are shown in the final rows.

Model (Split)	B cells	CD14+ Mono	CD4 T	CD8 T	Dendritic	FCGR3A+ Mono	Megakaryocytes	NK cells
CoupleVAE (All Genes)	0.867	0.961	0.735	0.826	0.828	0.925	0.866	0.898
scPreGAN (All Genes)	0.865	0.957	0.733	0.823	0.825	0.923	0.864	0.893
trVAE (All Genes)	0.862	0.950	0.729	0.821	0.818	0.923	0.864	0.890
scDist (All Genes)	0.852	0.947	0.729	0.822	0.813	0.911	0.862	0.893
scGen (All Genes)	0.862	0.955	0.726	0.811	0.818	0.918	0.849	0.879
CVAE (All Genes)	0.846	0.951	0.710	0.823	0.808	0.917	0.849	0.881
**CFM-GP (All Genes)**	**0.871**	**0.965**	**0.738**	**0.831**	**0.832**	**0.929**	**0.869**	**0.901**
CoupleVAE (Top 100 DEGs)	0.812	0.958	0.783	0.807	0.887	0.946	0.497	0.887
scPreGAN (Top 100 DEGs)	0.915	0.911	0.795	0.686	0.911	0.889	0.499	0.903
trVAE (Top 100 DEGs)	0.739	0.862	0.761	0.734	0.795	0.962	0.496	0.820
scDist (Top 100 DEGs)	0.733	0.914	0.686	0.766	0.738	0.854	0.551	0.800
scGen (Top 100 DEGs)	0.729	0.891	0.749	0.688	0.800	0.891	0.473	0.905
CVAE (Top 100 DEGs)	0.700	0.938	0.653	0.780	0.703	0.763	0.543	0.846
**CFM-GP (Top 100 DEGs)**	**0.932**	**0.994**	**0.849**	**0.828**	**0.942**	**0.984**	**0.608**	**0.973**
% Improvement (All Genes)	+0.45%	+0.45%	+0.41%	+0.74%	+0.54%	+0.43%	+0.41%	+0.40%
% Improvement (Top 100 DEGs)	+14.79%	+3.69%	+8.42%	+2.64%	+6.13%	+4.06%	+22.30%	+9.71%

Table S19: R^2 predictive accuracy across Statefate progenitor populations for All Genes and Top 100 DEGs. Rows represent models; columns indicate sorted cell populations. Percentage improvements for CFM-GP over CoupleVAE are shown in the final rows.

Model (Split)	Lin-Kit+Sca1+	Lin-Kit+Sca1-
CoupleVAE (All Genes)	0.891	0.924
scPreGAN (All Genes)	0.878	0.939
trVAE (All Genes)	0.905	0.952
scDist (All Genes)	0.887	0.862
scGen (All Genes)	0.796	0.889
CVAE (All Genes)	0.863	0.922
**CFM-GP (All Genes)**	**0.922**	**0.975**
CoupleVAE (Top 100 DEGs)	0.954	0.945
scPreGAN (Top 100 DEGs)	0.884	0.984
trVAE (Top 100 DEGs)	0.940	0.905
scDist (Top 100 DEGs)	0.894	0.906
scGen (Top 100 DEGs)	0.847	0.920
CVAE (Top 100 DEGs)	0.876	0.871
**CFM-GP (Top 100 DEGs)**	**0.962**	**0.996**
% Improvement (All Genes)	+3.49%	+5.50%
% Improvement (Top 100 DEGs)	+0.84%	+5.46%

Table S20: Maximum Mean Discrepancy (MMD) between predicted and real gene expression distributions for COVID-19 cell types across models. Lower values indicate better alignment. The rightmost column shows the improvement of CFM-GP over the baseline CoupleVAE.

Cell Type	CoupleVAE	scPreGAN	trVAE	scDist	scGen	CVAE	CFM-GP	Improvement
CD8 T	0.243	0.253	0.258	0.373	0.443	0.349	**0.214**	+11.82%
Macrophages	0.087	0.104	0.171	0.074	0.078	0.087	**0.015**	+83.29%
NK	0.082	0.124	0.072	0.248	0.188	0.201	**0.056**	+31.26%
Neutrophil	0.051	0.102	0.082	0.214	0.170	0.090	**0.021**	+58.27%
Secretory	0.132	0.101	0.242	0.240	0.287	0.265	**0.086**	+34.91%
mDC	0.187	0.184	0.225	0.149	0.332	0.230	**0.109**	+41.46%

Table S21: Maximum Mean Discrepancy (MMD) between predicted and real gene expression distributions for PBMC cell types across models. Lower values indicate better alignment. The rightmost column shows the percentage improvement of CFM-GP over CoupleVAE.

Cell Type	CoupleVAE	scPreGAN	trVAE	scDist	scGen	CVAE	CFM-GP	Improvement
B	0.0419	0.0776	0.0852	0.1498	0.0740	0.2450	**0.0182**	+56.61%
CD14+Mono	0.0647	0.0370	0.0486	0.1181	0.1668	0.1421	**0.0241**	+62.79%
CD4T	0.0775	0.0631	0.1299	0.1999	0.1242	0.0683	**0.0008**	+98.94%
CD8T	0.0834	0.0496	0.1269	0.2314	0.1892	0.1321	**0.0377**	+54.74%
Dendritic	0.0746	0.0585	0.1717	0.1073	0.2062	0.1494	**0.0364**	+51.24%
FCGR3A+Mono	0.0883	0.0575	0.1181	0.0603	0.2453	0.2746	**0.0152**	+82.84%
NK	0.0575	0.0856	0.1428	0.1320	0.2408	0.3164	**0.0370**	+35.68%

Table S22: Maximum Mean Discrepancy (MMD) between predicted and real gene expression distributions for glioblastoma samples across models. Lower values indicate better alignment. The rightmost column shows the percentage improvement of CFM-GP over CoupleVAE.

**Sample ID**	**CoupleVAE**	**scPreGAN**	**trVAE**	**scDist**	**scGen**	**CVAE**	**CFM-GP**	**Improvement**
PW029	0.128	0.091	0.213	0.113	0.379	0.467	**0.0085**	+93.39%
PW030	0.207	0.290	0.127	0.118	0.413	0.552	**0.0035**	+98.33%
PW032	0.154	0.114	0.282	0.142	0.084	0.384	**0.0051**	+96.72%
PW034	0.160	0.206	0.039	0.337	0.156	0.284	**0.0009**	+99.45%
PW036	0.113	0.180	0.223	0.125	0.326	0.0459	**0.0022**	+98.09%
PW052	0.198	0.305	0.272	0.187	0.0832	0.222	**0.0100**	+94.97%

Table S23: Maximum Mean Discrepancy (MMD) between predicted and real gene expression distributions for lupus cell types across models. Lower values indicate better alignment. The rightmost column shows the percentage improvement of CFM-GP over CoupleVAE.

**Cell Type**	**CoupleVAE**	**scPreGAN**	**trVAE**	**scDist**	**scGen**	**CVAE**	**CFM-GP**	**Improvement**
B cells	0.0299	0.1092	0.1898	0.0715	0.1768	0.1227	**0.0095**	+68.11%
CD14+ Mono	0.0138	0.1314	0.0284	0.2454	0.1718	0.0594	**0.0043**	+68.82%
CD4 T cells	0.0764	0.1566	0.1627	0.0344	0.2261	0.4308	**0.0023**	+96.95%
CD8 T cells	0.0374	0.2158	0.1245	0.1278	0.4205	0.0961	**0.0114**	+69.61%
Dendritic cells	0.1226	0.1255	0.2347	0.1921	0.1191	0.3478	**0.0571**	+53.39%
FCGR3A+ Mono	0.1032	0.0363	0.1400	0.3527	0.1726	0.0463	**0.0134**	+87.00%
Megakaryocytes	0.1811	0.2401	0.1456	0.4415	0.4202	0.5440	**0.1176**	+35.07%
NK cells	0.0656	0.1345	0.1059	0.2079	0.2295	0.2802	**0.0124**	+81.07%

Table S24: Maximum Mean Discrepancy (MMD) between predicted and real gene expression distributions for Statefate progenitor populations across models. Lower values indicate better alignment. The rightmost column shows the percentage improvement of CFM-GP over CoupleVAE.

**Cell Type**	**CoupleVAE**	**scPreGAN**	**trVAE**	**scDist**	**scGen**	**CVAE**	**CFM-GP**	**Improvement**
Lin-Kit+Sca1+	0.1245	0.0989	0.1284	0.1056	0.2682	0.1417	**0.0007**	+99.48%
Lin-Kit+Sca1-	0.0096	0.0421	0.0534	0.2408	0.4274	0.4879	**0.0017**	+82.37%

Table S25: Spearman rank correlation (ρ) between predicted and actual gene expression profiles for COVID-19 dataset. Higher values indicate better preservation of gene expression rankings. Rightmost column reports the improvement of CFM-GP over CoupleVAE.

**Cell Type**	**CoupleVAE**	**scPreGAN**	**trVAE**	**scDist**	**scGen**	**CVAE**	**CFM-GP**	**Improvement (%)**
CD8 T	0.455	0.360	0.324	0.308	0.181	0.460	**0.522**	+14.7%
Macrophages	0.538	0.617	0.449	0.405	0.387	0.288	**0.632**	+17.4%
NK	0.596	0.656	0.633	0.594	0.545	0.449	**0.689**	+15.6%
Neutrophil	0.494	0.544	0.389	0.293	0.521	0.316	**0.570**	+15.4%
Secretory	0.767	0.782	0.620	0.729	0.550	0.623	**0.805**	+5.0%
mDC	0.469	0.445	0.486	0.299	0.247	0.314	**0.561**	+19.7%

Table S26: Spearman rank correlation (ρ) between predicted and actual gene expression profiles for PBMC dataset. Higher values indicate better preservation of gene expression rankings. Rightmost column reports the improvement of CFM-GP over CoupleVAE.

**Cell Type**	**CoupleVAE**	**scPreGAN**	**trVAE**	**scDist**	**scGen**	**CVAE**	**CFM-GP**	**Improvement (%)**
B	0.684	0.698	0.535	0.703	0.552	0.571	**0.760**	+11.1%
CD14+Mono	0.515	0.507	0.458	0.416	0.343	0.530	**0.584**	+13.5%
CD4T	0.846	0.842	0.820	0.787	0.691	0.486	**0.879**	+3.9%
CD8T	0.579	0.658	0.622	0.547	0.618	0.534	**0.678**	+17.1%
Dendritic	0.546	0.535	0.488	0.583	0.532	0.521	**0.644**	+18.0%
FCGR3A+Mono	0.599	0.551	0.549	0.548	0.346	0.428	**0.628**	+4.8%
NK	0.619	0.581	0.548	0.575	0.448	0.399	**0.686**	+10.9%

Table S27: Spearman rank correlation (ρ) between predicted and actual gene expression profiles for glioblastoma patients. Higher values reflect stronger consistency in gene expression ordering. Rightmost column reports the improvement of CFM-GP over CoupleVAE.

Cell Type	CoupleVAE	scPreGAN	trVAE	scDist	scGen	CVAE	CFM-GP	Improvement (%)
PW029	0.896	0.804	0.899	0.872	0.932	0.717	**0.963**	+7.5%
PW030	0.882	0.824	0.863	0.787	0.763	0.903	**0.966**	+9.5%
PW032	0.903	0.798	0.743	0.895	0.810	0.646	**0.917**	+1.5%
PW034	0.932	0.875	0.920	0.918	0.864	0.743	**0.979**	+5.0%
PW036	0.910	0.958	0.957	0.943	0.915	0.756	**0.976**	+7.3%
PW052	0.861	0.864	0.864	0.883	0.786	0.686	**0.936**	+8.7%

Table S28: Spearman rank correlation (ρ) between predicted and actual gene expression profiles for lupus dataset. Higher values indicate better preservation of gene expression rankings. Rightmost column reports the improvement of CFM-GP over CoupleVAE.

Cell Type	CoupleVAE	scPreGAN	trVAE	scDist	scGen	CVAE	CFM-GP	Improvement (%)
B cells	0.608	0.597	0.547	0.474	0.546	0.542	**0.652**	+7.2%
CD14+ Monocytes	0.699	0.693	0.594	0.535	0.476	0.366	**0.714**	+2.1%
CD4 T cells	0.496	0.532	0.533	0.477	0.417	0.258	**0.562**	+13.3%
CD8 T cells	0.593	0.558	0.560	0.606	0.496	0.487	**0.633**	+6.7%
Dendritic cells	0.534	0.525	0.450	0.524	0.429	0.271	**0.580**	+8.6%
FCGR3A+ Monocytes	0.615	0.608	0.531	0.550	0.510	0.593	**0.633**	+2.9%
Megakaryocytes	0.478	0.540	0.431	0.332	0.523	0.420	**0.557**	+16.5%
NK cells	0.627	0.570	0.577	0.523	0.369	0.598	**0.657**	+4.8%

Table S29: Spearman rank correlation (ρ) between predicted and actual gene expression profiles for Statefate progenitor populations. Higher values indicate better preservation of gene expression rankings. Rightmost column reports the improvement of CFM-GP over CoupleVAE.

Cell Type	CoupleVAE	scPreGAN	trVAE	scDist	scGen	CVAE	CFM-GP	Improvement (%)
Lin-Kit+Sca1+	0.783	0.697	0.699	0.705	0.707	0.659	**0.805**	+2.8%
Lin-Kit+Sca1-	0.849	0.856	0.835	0.854	0.819	0.692	**0.898**	+5.8%

Table S30: Mean absolute error (MAE) across datasets. Values are summarized across cell types within each dataset (mean ± std; range shown as min–max).

**Dataset**	**# Cell Types**	**Mean MAE**	**Std**	**Min–Max**
COVID-19	6	0.0285	0.0146	0.0162–0.0529
PBMC IFN-β	7	0.0164	0.0052	0.0076–0.0227
Lupus	8	0.0721	0.0363	0.0426–0.1510
Glioblastoma	6	0.0815	0.0131	0.0638–0.0983
Statefate	2	0.0372	0.0054	0.0334–0.0410

Supplementary Figures

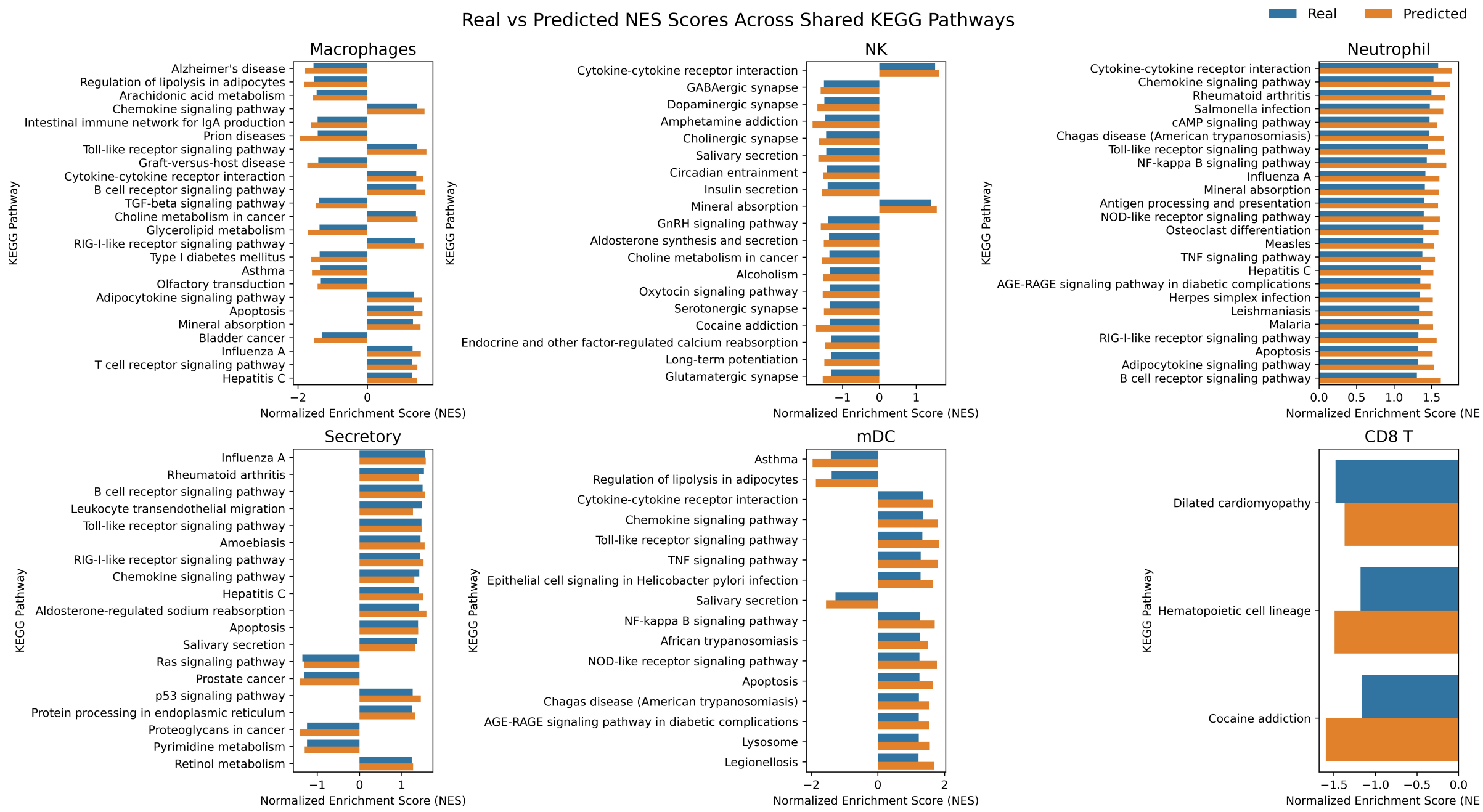

Figure S1: Pathway enrichment analysis for COVID-19 datasets comparing real and CFM-GP–predicted gene expression responses using normalized enrichment scores (NES). Top pathways enriched in predicted data are compared to those in real perturbed cells, and the degree of overlap reflects the biological consistency of the model outputs.

Table S31: Cross-species generalization performance measured by R^2 for CFM-GP and CoupleVAE. Higher values indicate better predictive accuracy. The rightmost column reports the percentage improvement of CFM-GP over CoupleVAE for each source-target pair.

**Source Species**	**Target Species**	**CFM-GP**	**CoupleVAE**	**Improvement (%)**
rat	rabbit	0.8275	0.8039	+2.94%
rat	pig	0.8268	0.7901	+4.64%
rat	mouse	0.8017	0.7414	+8.13%
rabbit	rat	0.8536	0.8571	−0.41%
rabbit	pig	0.8087	0.7928	+2.00%
rabbit	mouse	0.7770	0.7405	+4.93%
pig	rat	0.8543	0.8571	−0.33%
pig	rabbit	0.8134	0.8685	−6.35%
pig	mouse	0.7609	0.7389	+2.97%
mouse	rat	0.8439	0.8171	+3.29%
mouse	rabbit	0.7846	0.7511	+4.46%
mouse	pig	0.7869	0.7906	−0.47%

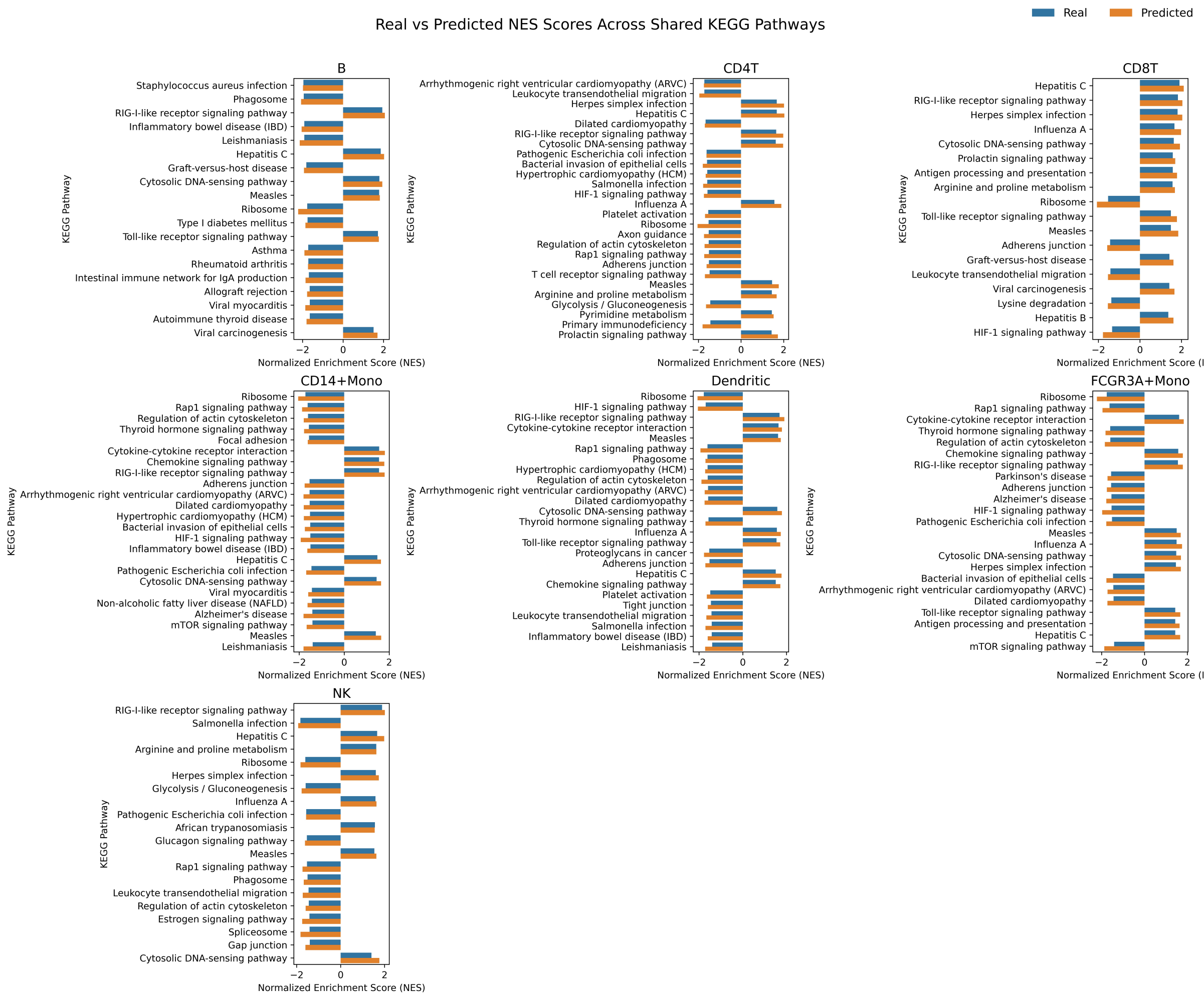

Figure S2: Pathway enrichment analysis for PBMC datasets comparing real and CFM-GP–predicted gene expression responses using normalized enrichment scores (NES). Top pathways enriched in predicted data are compared to those in real perturbed cells, and the degree of overlap reflects the biological consistency of the model outputs.

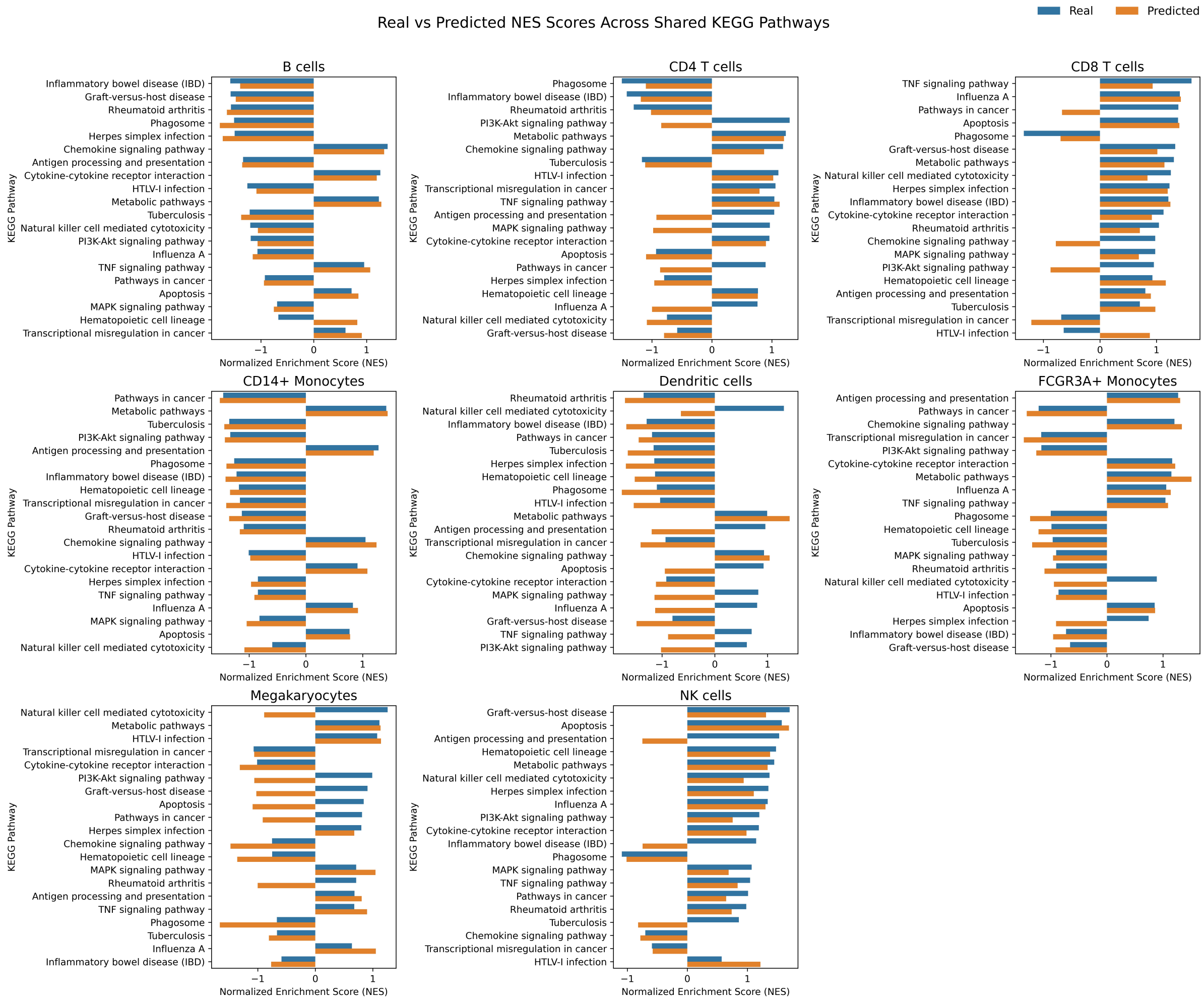

Figure S3: Pathway enrichment analysis for Lupus datasets comparing real and CFM-GP–predicted gene expression responses using normalized enrichment scores (NES). Top pathways enriched in predicted data are compared to those in real perturbed cells, and the degree of overlap reflects the biological consistency of the model outputs.

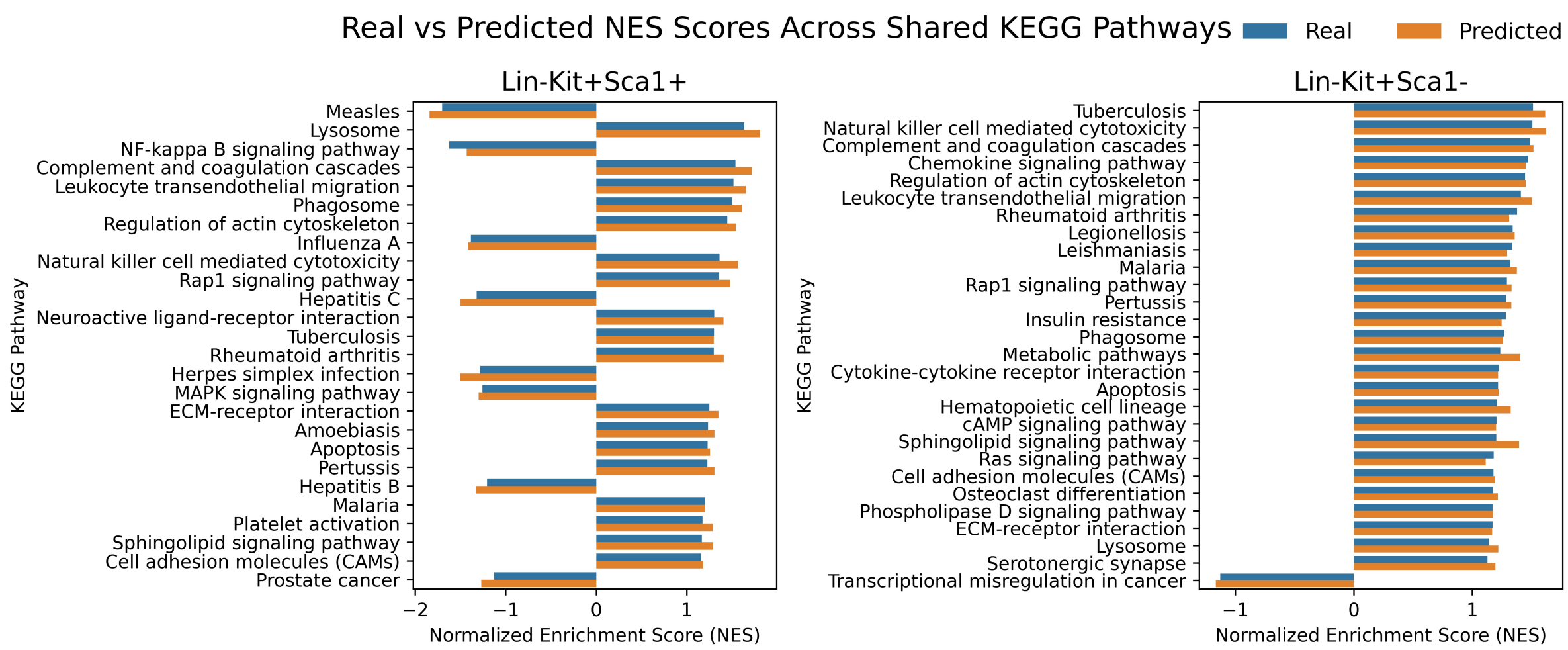

Figure S4: Pathway enrichment analysis for Statefate datasets comparing real and CFM-GP–predicted gene expression responses using normalized enrichment scores (NES). Top pathways enriched in predicted data are compared to those in real perturbed cells, and the degree of overlap reflects the biological consistency of the model outputs.

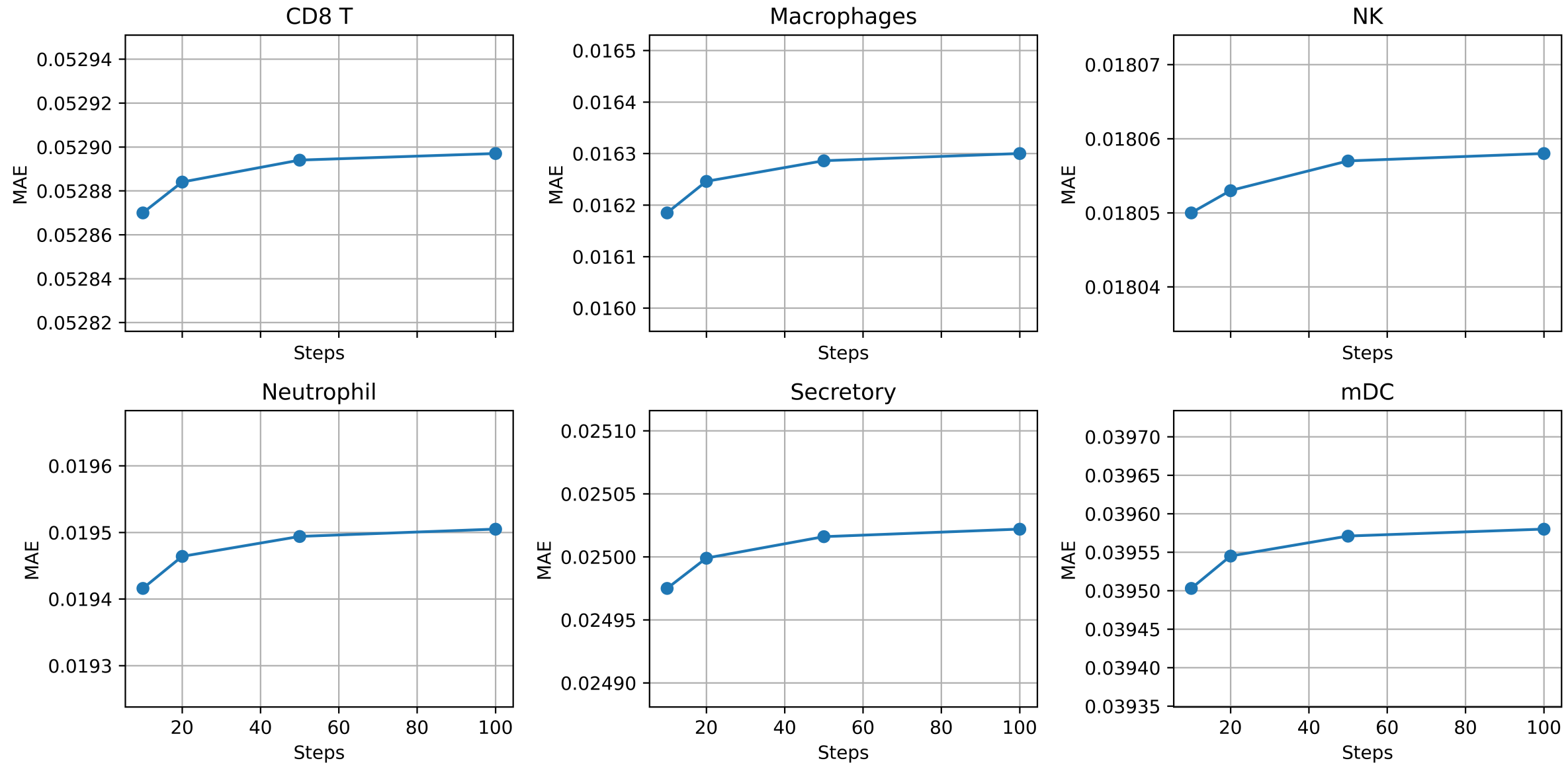

Figure S5: Mean absolute error (MAE) across integration steps (10–100) for the COVID-19 dataset. MAE remains stable across cell types and integration depths, indicating numerical robustness and consistent absolute prediction accuracy. Notably, the monotonic but minimal change in MAE indicates that numerical discretization error is negligible relative to model prediction error.

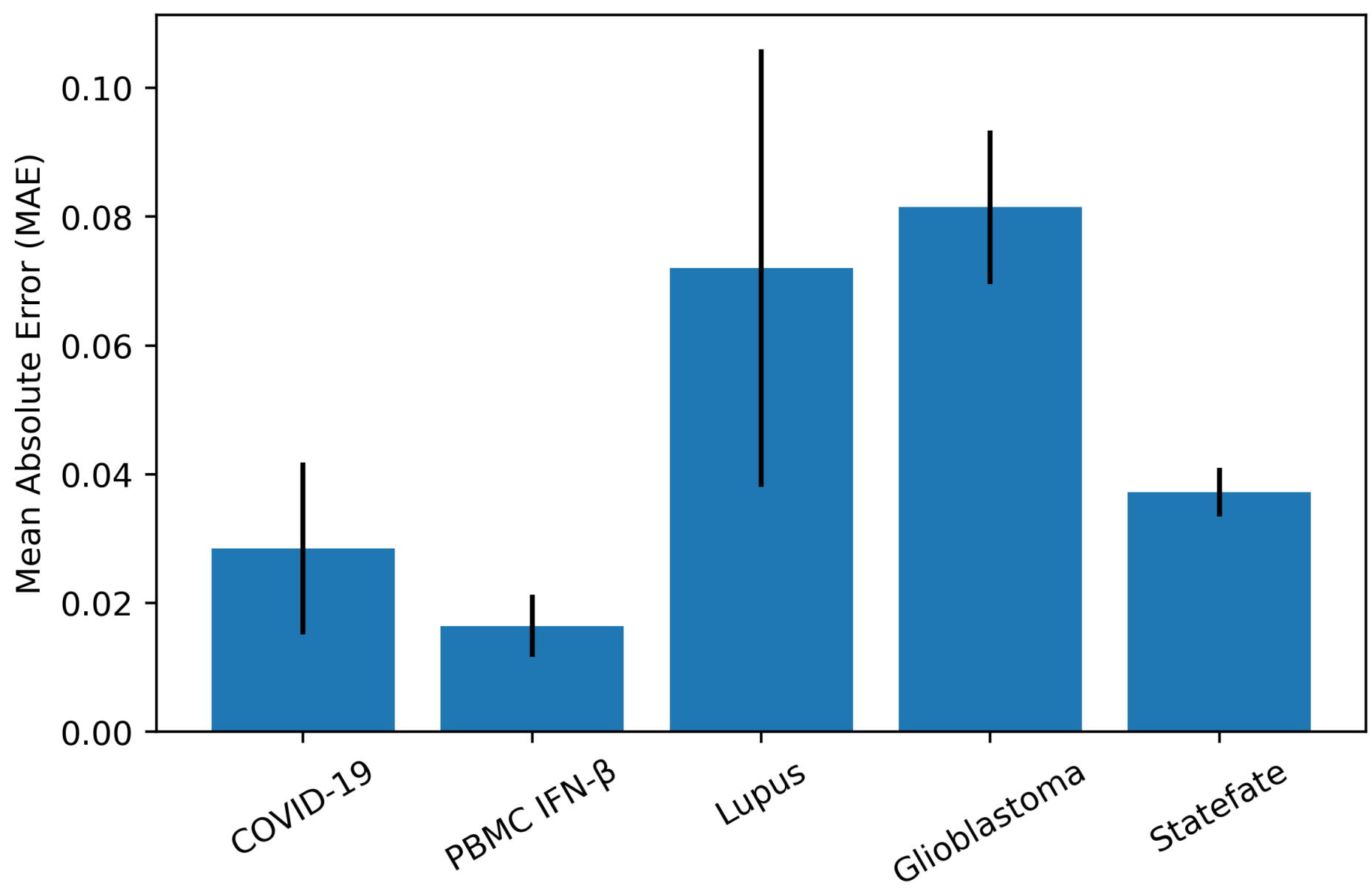

Figure S6: Mean absolute error (MAE) across datasets. Bars represent the mean MAE across cell types within each dataset, and error bars denote standard deviation. Consistent with improvements observed in R^2, MAE values remain low across all datasets, confirming that performance gains are not driven by variance inflation but reflect genuine reductions in absolute gene-level prediction error.

References

[1] Lotfollahi, M., Naghipourfar, M., Luecken, M. D., Khajavi, M., Büttner, M., Wagenstetter, M., Avsec, Ž., Gayoso, A., Yosef, N., Interlandi, M., et al. (2022) Mapping single-cell data to reference atlases by transfer learning. *Nature biotechnology,* **40**(1), 121–130.

[2] Zhao, W., Dovas, A., Spinazzi, E. F., Levitin, H. M., Banu, M. A., Upadhyayula, P., Sudhakar, T., Marie, T., Otten, M. L., Sisti, M. B., et al. (2021) Deconvolution of cell type-specific drug responses in human tumor tissue with single-cell RNA-seq. *Genome medicine,* **13**(1), 82.

[3] Kang, H. M., Subramaniam, M., Targ, S., Nguyen, M., Maliskova, L., McCarthy, E., Wan, E., Wong, S., Byrnes, L., Lanata, C. M., et al. (2018) Multiplexed droplet single-cell RNA-sequencing using natural genetic variation. *Nature biotechnology,* **36**(1), 89–94.

[4] Weinreb, C., Rodriguez-Fraticelli, A., Camargo, F. D., and Klein, A. M. (2020) Lineage tracing on transcriptional landscapes links state to fate during differentiation. *Science,* **367**(6479), eaaw3381.

[5] Kumar, S., Stecher, G., Suleski, M., and Hedges, S. B. (2017) TimeTree: a resource for timelines, timetrees, and divergence times. *Molecular biology and evolution,* **34**(7), 1812–1819.
